# Supplementary material for: Arsenal of nanobodies shows broad-spectrum neutralization against SARS-CoV-2 variants of concern in vitro and in vivo in hamster models
Source: Commun Biol. 2022 Sep 9;5:933. doi: 10.1038/s42003-022-03866-z (PMC9461429; doi:10.1038/s42003-022-03866-z)
Supplement: Supplementary file 1 — Supplementary Information [file 42003_2022_3866_MOESM1_ESM.pdf]

Supplementary Information for

Arsenal of nanobodies shows broad-spectrum neutralization against SARS-CoV-2 variants of concern *in vitro* and *in vivo* in hamster models

Martin A. Rossotti[1], Henk van Faassen[1], Anh T. Tran[1], Joey Sheff[1], Jagdeep K. Sandhu[2,3], Diana Duque[1], Melissa Hewitt[2], Xiaoxue Wen[1], Jegarubee Bavananthasivam[1], Saina Beitari[1], Kevin Matte[3], Geneviève Laroche[3], Patrick M. Giguère[3,4], Christian Gervais[5], Matthew Stuible[5], Julie Guimond[5], Sylvie Perret[5], Greg Hussack[1], Marc-André Langlois[3], Yves Durocher[5,6] & Jamshid Tanha[1,3*]

[1]Human Health Therapeutics Research Centre, Life Sciences Division, National Research Council Canada, 100 Sussex Drive, Ottawa, Ontario, Canada; [2]Human Health Therapeutics Research Centre, Life Sciences Division, National Research Council Canada, 1200 Montreal Road, Ottawa, Ontario, Canada; [3]Department of Biochemistry, Microbiology and Immunology, Faculty of Medicine, University of Ottawa, 451 Smyth Road, Ottawa, Ontario, Canada; [4]University of Ottawa Brain and Mind Research Institute, University of Ottawa, 451 Smyth Road, Ottawa, Ontario, Canada; [5]Human Health Therapeutics Research Centre, Life Sciences Division, National Research Council Canada, 6100 Royalmount Avenue, Montréal, Québec, Canada; [6]Département de biochimie et médecine moléculaire, Université de Montréal, Pavillon Roger-Gaudry, C.P. 6128, Succ. Centre-ville, Montréal, Québec, Canada

[*]Correspondence to: Jamshid Tanha, Ph.D., Human Health Therapeutics Research Centre, Life Sciences Division, National Research Council Canada, 100 Sussex Drive, Ottawa, Ontario, Canada, K1A 0R6, Tel: 613-990-7206; Email: Jamshid.Tanha@nrc-cnrc.gc.ca

This PDF file includes: (i) Supplementary Tables 1-9 and (ii) Supplementary Figures 1-23.

Other Supplementary Information for the manuscript includes: Supplementary Data 1.

Supplementary Table 1. Coronavirus spike glycoprotein and ACE2 fragments used in this study.

Description	Accession number	Source	Tag	Reference describing expression & purification
S1 (aa16-685) (Wuhan)	QHD43416.1[b]	in-house	FLAG, 6xHis	Akache et al.,2021[1]
S1 (aa16-685) (Wuhan)	QHD43416.1[b]	ACROBiosystems	AviTag, 6xHis	na
S1 (aa16-685) (Wuhan)	QHD43416.1[b]	ACROBiosystems	Human IgG1 Fc	na
NTD (aa16-305) (Wuhan)	QHD43416.1[b]	in-house	FLAG, 6xHis	Akache et al.,2021[1]
RBD/SD1 (aa319-591) (Wuhan)	QHD43416.1[b]	in-house	Human IgG1 Fc	Wrapp et al., 2020[2]
RBD/SD1 (aa319-591) (Wuhan)	QHD43416.1[b]	in-house	6xHis	Wrapp et al., 2020[2]
RBD_short (aa331-521) (Wuhan)	QHD43416.1[b]	in-house	6xHis	Akache et al.,2021[1]
RBD (aa319-541) (Wuhan)	QHD43416.1[b]	in-house	FLAG-6xHis (N-term), E5 (C-term)	Akache et al.,2021[1]
RBD_α (aa319-541) (B.1.1.7)	QHD43416.1[b]	in-house	FLAG, 6xHis	Colwill et al., 2021[3]
RBD_β (aa319-541) (B.1.351)	QHD43416.1[b]	in-house	FLAG, 6xHis	Colwill et al., 2021[3]
RBD_Wuhan (aa319-541)	QHD43416.1[b]	ACROBiosystems	AviTag, 6xHis	na
RBD_SARS-CoV (aa306-527)	AAX16192.1[b]	In-house	FLAG, 6xHis	Colwill et al., 2021[3]
S2 (aa686-1208) (Wuhan)	QHD43416.1[b]	in-house	FLAG, 6xHis	Akache et al.,2021[1]
Swine deltaCoV S[a]	AIH06857.1[b]	in-house	FLAG-Dual Strep-6xHis	Galipeau et al., 2021[4]
Avian_IBV S[a]	AAP92675.1[b]	in-house	FLAG-Dual Strep-6xHis	Galipeau et al., 2021[4]
Pangolin CoV S[a]	QIA48632.1[b]	in-house	FLAG-Dual Strep-6xHis	Galipeau et al., 2021[4]
Hedgehog CoV HKU31 S[a]	QGA70702.0[b]	in-house	FLAG-Dual Strep-6xHis	Galipeau et al., 2021[4]
Bat CoV HKU9 S[a]	YP_001039971.1[c]	in-house	FLAG-Dual Strep-6xHis	Galipeau et al., 2021[4]
Bat SARS like CoV-WIV1 S[a]	AGZ48828.1[b]	in-house	FLAG-Dual Strep-6xHis	Galipeau et al., 2021[4]
Bat 229E-related CoV S[a]	APD51507.1[b]	in-house	FLAG-Dual Strep-6xHis	Galipeau et al., 2021[4]
Bat CoV 512 S[a]	YP_001351684.1[c]	in-house	FLAG-Dual Strep-6xHis	Galipeau et al., 2021[4]
Bat SARS like CoV[a]	ATO98157.1[b]	in-house	FLAG-Dual Strep-6xHis	Galipeau et al., 2021[4]
Civet SARS-CoV[1] S[a]	AAU04646.1[b]	in-house	FLAG-Dual Strep-6xHis	Galipeau et al., 2021[4]
Human MERS-CoV S[a]	AGH58717.1[b]	in-house	FLAG-Dual Strep-6xHis	Galipeau et al., 2021[4]
Human CoV-NL63 S	APF29071.1[b]	Sino Biological	6xHis	na
Human CoV-OC43 S[a]	AGT51431.1[b]	in-house	FLAG-Dual Strep-6xHis	Galipeau et al., 2021[4]
Human CoV-HKU1 S	Q0ZME7.1[d]	Sino Biological	6xHis	na

Human CoV-229E S[a]	AAK32191.1[b]	Sino Biological	6xHis	na
Human SARS-CoV S[a]	AAP13442.1[b]	in-house	FLAG-Dual Strep-6xHis	Galipeau et al., 2021[4]
Human SARS-CoV-2_Wuhan S[a] (SmT1)	QHD43416.1[b]	in-house	FLAG, 6xHis	Colwill et al., 2021[3]
Human SARS-CoV-2_α (B.1.351) S[a]	-	in-house	FLAG, 6xHis	Galipeau et al., 2021[4]
Human SARS-CoV-2_β (B. 1.1.7) S[a]	-	in-house	FLAG, 6xHis	Galipeau et al., 2021[4]
Human SARS-CoV-2_γ (P.1) S[a]	-	in-house	none	Galipeau et al., 2021[4]
Human SARS-CoV-2_Δ (B.1.617.2) S[a]	-	in-house	FLAG, 6xHis	Stuible et al., 2021[5]
Human SARS-CoV-2_κ (B.1.617.1) S[a]	-	in-house	FLAG, 6xHis	Stuible et al., 2021[5]
Human SARS-CoV-2_Ο (B.1.1.529) S[a]	-	in-house	FLAG, 6xHis	Sulea et al., 2022[6]
Human ACE2 (aa18-740)	Q9BYF1-1[d]	ACROBiosystems	Human IgG1 Fc	na
Human ACE2 (aa18-615)	Q9BYF1-1[d]	in-house	6xHis	Wrapp et al., 2020[2]

[a]Proteins are C-terminally fused to the resistin trimerization domain. [b]GenBank; [c]NCBI; [d]UniProt. na, not applicable

Supplementary Table 2. Kinetic and equilibrium constants for V_HHs binding to SARS-CoV-2 Wuhan spike glycoprotein fragments.

V_HH/ACE2	RBD_short[a]			RBD/SD1[a]			S[a]			S1[a]			S2[a]		
	k_a (1/Ms)	k_d (1/s)	K_D (M)	k_a (1/Ms)	k_d (1/s)	K_D (M)	k_a (1/Ms)	k_d (1/s)	K_D (M)	k_a (1/Ms)	k_d (1/s)	K_D (M)	k_a (1/Ms)	k_d (1/s)	K_D (M)
**RBD-specific V_HH**															
1d	9.26E+05	3.76E-03	4.06E-09	5.62E+05	1.18E-03	2.10E-09	1.06E+06	1.17E-03	1.10E-09	8.67E+05	1.14E-03	1.32E-09	-	-	-
02	2.01E+06	1.73E-03	8.60E-10	1.83E+06	1.41E-03	7.73E-10	2.14E+06	1.41E-03	6.61E-10	2.10E+06	1.38E-03	6.59E-10	-	-	-
03	-	-	-	1.66E+05	2.55E-04	1.53E-09	2.50E+05	3.34E-04	1.34E-09	2.41E+05	2.63E-04	1.09E-09	-	-	-
04	1.40E+06	2.02E-02	1.45E-08	1.53E+06	1.92E-02	1.25E-08	1.97E+06	1.98E-02	1.00E-08	1.61E+06	1.80E-02	1.12E-08	-	-	-
05	2.97E+06	7.13E-03	2.40E-09	2.51E+06	5.85E-03	2.33E-09	4.33E+06	7.43E-03	1.72E-09	4.03E+06	8.06E-03	2.00E-09	-	-	-
06	9.83E+03	6.87E-03	6.99E-07	5.94E+04	4.15E-03	6.99E-08	3.02E+04	4.69E-03	1.55E-07	1.04E+05	1.33E-02	1.29E-07	-	-	-
07	4.25E+05	4.54E-04	1.07E-09	3.18E+05	2.84E-04	8.94E-10	3.50E+05	4.03E-04	1.15E-09	3.01E+05	3.08E-04	1.02E-09	-	-	-
10	3.79E+05	1.01E-04	2.66E-10	3.51E+05	9.32E-05	2.66E-10	4.83E+05	9.27E-05	1.92E-10	4.48E+05	9.54E-05	2.13E-10	-	-	-
11	-	-	-	8.92E+05	2.26E-04	2.53E-10	1.21E+06	4.73E-05	3.91E-11	1.08E+06	1.80E-04	1.67E-10	-	-	-
12	5.33E+05	4.78E-05	8.97E-11	7.60E+05	3.69E-05	4.86E-11	nd								
14	2.98E+05	1.11E-03	3.71E-09	2.61E+05	9.44E-04	3.61E-09	5.28E+05	1.64E-03	3.10E-09	3.19E+05	1.12E-03	3.49E-09	-	-	-
15	9.01E+05	2.89E-04	3.21E-10	6.82E+05	2.33E-04	3.42E-10	7.06E+05	2.21E-04	3.13E-10	6.75E+05	2.25E-04	3.33E-10	-	-	-
17	9.53E+05	3.48E-04	3.65E-10	5.67E+05	2.24E-04	3.95E-10	6.59E+05	9.79E-05	1.49E-10	6.29E+05	9.66E-05	1.53E-10	-	-	-
18	3.35E+05	1.80E-03	5.37E-09	2.68E+05	1.97E-04	7.36E-10	3.95E+05	1.52E-04	3.84E-10	3.08E+05	1.65E-04	5.36E-10	-	-	-
20	2.67E+06	2.10E-02	7.84E-09	1.43E+06	1.23E-02	8.61E-09	2.37E+06	1.59E-02	6.73E-09	1.96E+06	1.89E-02	9.63E-09	-	-	-
MRed04	1.58E+06	1.08E-02	6.86E-09	6.04E+05	1.51E-03	2.51E-09	1.39E+06	1.50E-03	1.09E-09	1.09E+06	1.83E-03	1.68E-09	-	-	-
MRed05	4.68E+05	6.33E-04	1.36E-09	6.50E+05	4.96E-04	7.63E-10	nd								
**NTD-specific V_HH**															
SR01	-	-	-	-	-	-	2.77E+06	1.23E-03	4.45E-10	3.63E+06	1.58E-03	4.37E-10	-	-	-
SR02	-	-	-	-	-	-	9.67E+05	5.71E-04	5.90E-10	1.05E+06	5.55E-04	5.30E-10	-	-	-
SR03	-	-	-	-	-	-	1.01E+06	1.02E-03	1.01E-09	9.05E+05	1.03E-03	1.13E-09	-	-	-
SR04	-	-	-	-	-	-	2.39E+06	3.35E-04	1.40E-10	2.18E+06	4.93E-04	2.26E-10	-	-	-
SR13	-	-	-	-	-	-	1.83E+06	4.81E-03	2.62E-09	2.25E+06	5.45E-03	2.43E-09	-	-	-
SR16	-	-	-	-	-	-	6.57E+05	1.20E-03	1.82E-09	7.39E+05	1.15E-03	1.55E-09	-	-	-

MRed03	-	-	-	-	-	-	1.57E+05	8.14E-05	5.20E-10	2.76E+05	6.15E-05	2.23E-10	-	-	-
MRed06	-	-	-	-	-	-	1.85E+05	8.87E-04	4.80E-09	3.71E+05	1.57E-03	4.22E-09	-	-	-
MRed07	-	-	-	-	-	-	1.60E+06	3.78E-04	2.36E-10	1.13E+06	4.27E-04	3.78E-10	-	-	-
**S2-specific V_HH**															
S2A3	-	-	-	-	-	-	8.40E+04	1.30E-04	1.55E-09	-	-	-	4.60E+04	1.03E-04	2.23E-09
S2A4	-	-	-	-	-	-	3.49E+04	4.46E-04	1.28E-08	-	-	-	2.81E+04	4.14E-04	1.47E-08
S2F3	-	-	-	-	-	-	1.56E+05	4.73E-04	3.03E-09	-	-	-	1.01E+05	6.29E-04	6.22E-09
S2G3	-	-	-	-	-	-	1.62E+05	6.07E-04	3.74E-09	-	-	-	1.47E+05	6.27E-04	4.28E-09
S2G4	-	-	-	-	-	-	8.93E+05	2.07E-04	2.32E-10	-	-	-	9.25E+05	3.82E-04	4.13E-10
MRed11	-	-	-	-	-	-	2.34E+04	4.18E-04	1.78E-8	-	-	-	4.09E+04	2.13E-04	5.21E-09
MRed18	-	-	-	-	-	-	2.02E+05	1.53E-03	7.56E-09	-	-	-	1.52E+05	1.03E-03	6.80E-09
MRed19	-	-	-	-	-	-	1.59E+05	7.99E-04	5.01E-09	-	-	-	1.02E+05	8.40E-04	8.20E-09
MRed20	-	-	-	-	-	-	1.60E+05	1.46E-05	9.18E-11	-	-	-	1.13E+05	3.38E-05	2.99E-10
MRed22	-	-	-	-	-	-	3.47E+05	1.76E-04	5.06E-10	-	-	-	2.70E+05	2.84E-04	1.05E-09
MRed25	-	-	-	-	-	-	1.12E+05	1.15E-04	1.02E-09	-	-	-	1.22E+05	1.90E-04	1.56E-09
**Reference**															
VHH-72[b]	8.64E+05	4.22E-01	4.89E-07	6.67E+05	1.34E-01	2.00E-07	1.10E+06	1.56E-01	1.42E-07	9.40E+05	1.46E-01	1.56E-07	-	-	-
ACE-2[b]	5.92E+04	1.35E-02	2.28E-07	3.71E+04	1.18E-02	3.17E-07	6.02E+04	9.96E-03	1.65E-07	6.21E+04	1.24E-02	2.00E-07	-	-	-
NRCsdAb022[b]	-	-	-	-	-	-	-	-	-	-	-	-	-	-	-

[a]For any given V_HH, K_D values across different spike fragments were essentially consistent, except for V_HH 03/11 and 06/18 which respectively showed no or ~10-fold lower binding to RBD_short compared to RBD/SD1 and S1. Lack of V_HH binding for certain spike fragments are consistent with V_HHs' subunit/domain specificities. Binding parameters were determined by flowing monomeric V_HHs over sensorchip surfaces immobilized with various spike fragments, except for V_HH 12 and MRed05, which were obtained by flowing monomeric RBDs over V_HH-Fc-captured surfaces (for V_HH 12, RBD [aa319-541] instead of RBD/SD1 was used). Dashes indicate lack of binding. See Supplementary Table 1 for the primary structures of spike glycoprotein fragments. [b]ACE2-H_6 and VHH-72[7], positive controls, EGFR-specific V_HH NRCsdAb022[8], negative control.

Supplementary Table 3. ELISA data for the binding of V_HH-Fcs to SARS-CoV-2 Wuhan spike glycoprotein fragments.

V_HH-Fc[a]	EC_{50}app[b] (nM)				Domain specificity
	S	S1	NTD	RBD	
SR01	0.1	0.2	0.2	-	NTD
SR02	0.1	0.1	0.3	-	NTD
SR03	0.2	0.2	0.2	-	NTD
SR04	0.2	0.3	0.2	-	NTD
SR13	0.4	0.4	0.2	-	NTD
SR16	0.6	2.7	0.4	-	NTD
MRed03	0.2	0.5	0.7	-	NTD
MRed06	0.4	0.6	0.4	-	NTD
MRed07	1.2	1.5	0.8	-	NTD
02	0.1	0.1	-	0.2	RBD

[a]S1-specific V_HHs that did not bind to RBD by SPR (Supplementary Table 2) were tested for specificity against recombinant NTD domain (Supplementary Table 1). The RBD-specific V_HH 02 internal control gave the expected binding specificity profile; [b]EC_{50}app, apparent EC_{50}. Dashes indicate lack of binding.

Supplementary Table 4. Kinetic and equilibrium dissociation constants for the binding of V_HHs to SARS-CoV-2 Wuhan, SARS-CoV-2 Alpha, SARS-CoV-2 Beta and SARS-CoV spike glycoprotein fragments.

V_HH/ACE2	SARS-CoV-2 Wuhan[a]			SARS-CoV-2 Alpha[a]			SARS-CoV-2 Beta[a]			SARS-CoV[a]		
	k_a (1/Ms)	k_d (1/s)	K_D (M)	k_a (1/Ms)	k_d (1/s)	K_D (M)	k_a (1/Ms)	k_d (1/s)	K_D (M)	k_a (1/Ms)	k_d (1/s)	K_D (M)
**RBD-specific V_HH**												
1d	1.39E+06	1.05E-03	7.50E-10	1.13E+06	1.02E-03	9.07E-10	8.12E+05	9.56E-04	1.18E-09	-	-	-
02	2.04E+06	1.27E-03	6.24E-10	1.64E+06	2.22E-02	1.36E-08	-	-	-	-	-	-
03	1.16E+05	1.81E-04	1.56E-09	1.03E+05	1.54E-04	1.49E-09	5.54E+04	2.26E-04	4.08E-09	-	-	-
04	1.84E+06	1.88E-02	1.02E-08	1.67E+06	1.96E-02	1.17E-08	-	-	-	-	-	-
05	2.76E+06	7.09E-03	2.57E-09	2.28E+06	2.61E-02	1.14E-08	-	-	-	-	-	-
06	2.05E+04	4.56E-03	2.23E-07	2.05E+04	4.68E-03	2.29E-07	2.48E+04	6.14E-03	2.48E-07	-	-	-
07	3.78E+05	3.55E-04	9.39E-10	3.39E+05	3.78E-04	1.12E-09	3.05E+05	3.21E-04	1.05E-09	1.20E+05	1.46E-03	1.22E-08
10	5.81E+05	1.16E-04	1.99E-10	4.84E+05	1.02E-04	2.10E-10	2.70E+05	2.62E-03	9.73E-09	-	-	-
11	9.64E+05	1.71E-05	1.77E-11	9.28E+05	1.59E-05	1.71E-11	7.57E+05	1.72E-05	2.27E-11	1.93E+06	2.70E-05	1.40E-11
12[a]	7.47E+05	3.50E-05	4.69E-11	9.47E+05	4.38E-05	4.63E-11	9.70E+05	3.89E-05	4.01E-11	3.76E+05	1.01E-03	2.69E-09
14	3.89E+05	1.01E-03	2.60E-09	3.75E+05	9.17E-04	2.44E-09	-	-	-	-	-	-
15	6.91E+05	2.22E-04	3.21E-10	6.37E+05	1.94E-04	3.05E-10	1.48E+05	3.28E-03	2.22E-08	-	-	-
17	6.14E+05	9.45E-05	1.54E-10	6.14E+05	7.64E-05	1.25E-10	1.14E+06	5.88E-03	5.14E-09	-	-	-
18	2.95E+05	9.39E-05	3.18E-10	2.78E+05	9.79E-05	3.53E-10	2.76E+05	1.01E-04	3.65E-10	-	-	-
20	2.64E+06	1.16E-02	4.39E-09	2.48E+06	1.23E-02	4.97E-09	2.08E+06	1.14E-02	5.47E-09	-	-	-
MRed04	1.51E+06	1.31E-03	8.63E-10	1.34E+06	1.22E-03	9.09E-10	1.14E+06	1.23E-03	1.07E-09	3.23E+05	9.70E-02	3.00E-07
MRed05[a]	5.62E+05	5.09E-04	9.05E-10	5.73E+05	1.76E-04	3.07E-10	6.12E+05	5.44E-04	8.88E-10	-	-	-
**NTD-specific V_HH**												
SR01	1.43E+06	8.05E-04	5.64E-10	1.01E+06	5.94E-04	5.91E-10	1.09E+06	2.21E-04	2.02E-10	6.81E+05	1.05E-04	1.54E-10
SR02	4.98E+06	6.69E-04	1.35E-10	4.54E+06	2.54E-04	5.59E-11	4.19E+06	6.28E-04	1.50E-10	-	-	-
SR03	6.70E+05	1.13E-03	1.69E-09	5.55E+05	9.55E-04	1.72E-09	5.64E+05	1.40E-03	2.49E-09	-	-	-
SR04	3.68E+06	5.15E-04	1.40E-10	2.80E+06	7.50E-04	2.67E-10	2.24E+06	7.23E-04	3.24E-10	-	-	-
SR13	1.06E+06	3.76E-03	3.56E-09	5.92E+05	3.44E-03	5.82E-09	5.71E+05	4.01E-03	7.02E-09	-	-	-
SR16	4.88E+05	9.57E-04	1.96E-09	4.04E+05	6.33E-04	1.57E-09	3.95E+05	1.01E-03	2.57E-09	-	-	-
MRed03	2.37E+05	1.21E-04	5.08E-10	2.71E+05	9.89E-05	3.64E-10	2.13E+05	1.43E-04	6.72E-10	-	-	-

MRed06	1.92E+05	9.96E-04	5.19E-09	2.27E+05	1.30E-03	5.72E-09	1.39E+05	1.01E-03	7.24E-09	-	-	-
MRed07	4.58E+06	4.81E-04	1.05E-10	3.90E+06	1.03E-03	2.64E-10	2.38E+06	5.52E-04	2.31E-10	-	-	-
**S2-specific V_HH**												
S2A3	9.83E+04	5.51E-05	5.61E-10	8.70E+04	1.89E-04	2.18E-09	6.69E+04	5.71E-05	8.53E-10	-	-	-
S2A4	3.49E+04	4.46E-04	1.28E-08	2.48E+05	2.36E-03	9.52E-09	5.88E+4	8.98E-4	1.53E-8	-	-	-
S2F3	1.56E+05	4.73E-04	3.03E-09		+			+		2.82E+05	1.39E-03	4.91E-09
S2G3	3.24E+05	6.06E-04	1.87E-09	2.98E+05	5.30E-04	1.78E-09	3.04E+05	5.63E-04	1.85E-09	1.87E+05	8.00E-04	4.27E-09
S2G4	8.93E+05	2.07E-04	2.32E-10	1.56E+06	2.92E-04	1.87E-10		+		9.20E+05	7.35E-04	7.99E-10
MRed11	4.57E+04	2.83E-04	6.20E-09	3.11E+04	4.26E-04	1.37E-08	4.54E+04	2.82E-04	6.21E-09	-	-	-
MRed18	1.97E+05	1.19E-03	6.03E-09	3.82E+05	4.93E-03	1.29E-08	3.69E+05	2.39E-03	6.48E-09	3.00E+05	6.77E-03	2.25E-08
MRed19	1.31E+05	1.18E-03	9.07E-09	1.83E+05	3.70E-03	2.02E-08	1.29E+05	1.04E-03	8.07E-09	2.49E+05	6.14E-03	2.46E-08
MRed20	1.60E+05	1.46E-05	9.18E-11	2.78E+05	1.53E-04	5.50E-10	1.39E+05	6.33E-05	4.55E-10	3.80E+05	4.06E-03	1.07E-08
MRed22	3.47E+05	1.76E-04	5.06E-10	8.96E+05	2.20E-04	2.46E-10		+		-	-	-
MRed25	1.12E+05	1.15E-04	1.02E-09	1.02E+06	2.89E-04	2.83E-10	2.05E+06	3.29E-03	1.60E-09	2.18E+04	5.01E-05	2.29E-09
**Control**												
ACE2-H_6[b]	6.38E+04	9.79E-03	1.53E-07	8.52E+04	1.56E-03	1.83E-08	3.66E+04	4.78E-03	1.31E-07	1.11E+05	3.89E-02	3.51E-07
VHH-72[b]	1.23E+06	1.06E-01	8.62E-08	1.05E+06	1.01E-01	9.60E-08	8.01E+05	9.92E-02	1.24E-07	1.01E+06	6.56E-03	6.52E-09
NRCsdAb 022[b]	-	-	-	-	-	-	-	-	-	-	-	-

[a]Binding parameters were determined by flowing monomeric V_HHs over sensorchip surfaces immobilized with S, except for V_HH 12 and MRed05, which were obtained by flowing monomeric RBDs (aa319-541 [SARS-CoV-2]; aa306-527 [SARS-CoV]) over V_HH-Fc-captured surfaces. Dashes indicate lack of binding. +, V_HH bound, but poor fitting precluded k_a, k_d, and K_D determinations. See Supplementary Table 1 for the primary structures of spike glycoprotein fragments; [b]ACE2-H_6 and VHH-72[7], positive controls, EGFR-specific V_HH NRCsdAb022[8] negative control.

Supplementary Table 5. Summary of V_HH stability data.

V_HH	Temperature-induced denaturation	GdnHCl-induced denaturation		
	T_m (°C)	ΔG^0 (kJ/mol)	C_m (M)	*m* (kJ/M*mol)
1d	65.5	27.1	1.8	15.4
02	67.3	31.3	1.9	16.9
03	79.5	nd	nd	nd
04	74.9	30.9	2.1	14.8
05	70.1	37.4	2.2	16.7
06	71.9	44	2.3	18.7
07	79.8	31.3	2.3	13.6
10	68.8	35.8	2.1	17.1
11	60.4	21.4	1.6	13.1
12	65.2	nd	nd	nd
14	68.9	27.5	2.2	12.3
15	76.9	35.9	2.2	16
17	73.3	35	2.6	13.6
18[a]	67.3	nd	0.3	nd
20	76.1	nd	nd	nd
MRed04	71.3	24.6	2.0	12.1
MRed05	69.7	nd	nd	nd
SR01	77.4	nd	nd	nd
SR02	63.4	nd	nd	nd
SR03	76.3	53.4	3.1	17.5
SR04	66.5	30.5	1.9	15.9
SR13	69.7	28.5	2.0	14
SR16	59.6	nd	nd	nd
S2A3	61.2	nd	nd	nd
S2A4	70.4	29.5	1.9	15.4
S2F3	64.6	nd	nd	nd
S2G3	70.4	nd	nd	nd
S2G4	69.3	28.4	2.2	12.9
MRed03	79.8	25.4	2.5	10.3
MRed06	59.1	nd	nd	nd
MRed07	68.4	26	2.2	12.1
MRed11	65.1	nd	nd	nd
MRed18	72.5	nd	nd	nd
MRed19	69.1	nd	nd	nd
MRed20	77.1	43.1	2.2	19.8
MRed22	72.5	31.3	2.2	14.3
MRed25	70.5	25.9	2.4	10.7
VHH-72	73	30.9	2.3	13.6

[a]Lack of a lower plateau due to an immediate melting upon exposure to the denaturant did not allow for a reliable curve fitting, hence ΔG^0 and *m* values for V_HH 18 could not be obtained. nd, not determined. Data were used to construct Fig. 3b and Fig. 3c.

Supplementary Table 6. Stability of V_HHs against aerosolization.

V_HH	Recovery (%)[a]	Soluble aggregates (%)[b]		ΔSoluble agg.[c]	Visible aggregates
		Pre-aerosolization	Post-aerosolization		
1d	83	2	4	2	No
02	89	2	2	0	No
03	81	2	1	-1	No
04	62	6	5	-1	Yes
05	89	2	5	3	No
06	51	7	5	-2	Yes
07	75	2	3	1	No
10	83	5	5	0	No
11	24	6	5	-1	Yes
14	55	6	6	0	Yes
15	69	4	5	1	Yes
17	85	5	6	1	No
18	99	9	5	-4	No
20	97	2	1	-1	No
SR03	43	3	11	8	Yes
SR04	52	5	3	-2	Yes
SR13	83	4	6	2	No
S2A4	84	7	11	4	No
S2G4	91	3	6	3	No
MRed03	96	3	2	-1	No
MRed04	59	4	4	0	Yes
MRed07	90	10	3	-7	No
MRed11	89	4	5	1	No
MRed18	96	3	10	7	No
MRed19	87	5	9	4	No
MRed20	76	2	18	16	No
MRed22	86	3	9	6	No
MRed25	44	3	3	0	Yes
VHH-72	78	1	14	13	No

[a]% recovery was determined as the proportion of a V_HH that remained monomer following aerosolization. Data were used to construct Fig. 3e (left panel) and Fig. 3e (middle panel); [b]% soluble aggregate was determined as the proportion of a V_HH that gave elution volume(s) smaller than that for the monomeric V_HH fraction; [c]ΔSoluble agg. = "Post-aerosolization" – "Pre-aerosolization".

Supplementary Table 7. V$_H$H binding data obtained by tandem SPR SVNAs against surface-immobilized SARS-CoV-2 S.

Summary orientation #1: V$_H$H followed by V$_H$H + ACE2							Summary orientation #2: ACE2 followed by ACE2 + V$_H$H						
**Cycle**	**Solution 1**	**Solution 2**	**End 1st inj[a] (RU)**	**End 2nd inj[a] (RU)**	**inj[a] 2-1 (ΔRU)**	**Blocker**	**Cycle**	**Solution 1**	**Solution 2**	**End 1st inj[a] (RU)**	**End 2nd inj[a] (RU)**	**inj[a] 2-1 (ΔRU)**	**Blocker**
1	Buffer	ACE2	-3.1	56	59.1	**No**	1	-	-	-	-	-	**-**
2	1d	1d+ACE2	26.3	40	13.7	**Yes**	2	ACE2	1d+ACE2	57.1	56.4	-0.7	**Yes**
3	02	02+ACE2	33	39.3	6.3	**Yes**	3	ACE2	02+ACE2	55.8	59.6	3.8	**Yes**
4	03	03+ACE2	18.1	82.3	64.2	**No**	4	ACE2	03+ACE2	52.8	75.3	22.5	**No**
5	04	04+ACE2	41.4	95.8	54.4	**No**	5	ACE2	04+ACE2	52.4	98.8	46.4	**No**
6	05	05+ACE2	36.9	46.8	9.9	**Yes**	6	ACE2	05+ACE2	52.2	60.5	8.3	**Yes**
7	06	06+ACE2	38.5	92.2	53.7	**No**	7	ACE2	06+ACE2	52.3	94.4	42.1	**No**
8	07	07+ACE2	19.1	37.5	18.4	**Yes**	8	ACE2	07+ACE2	52	59.1	7.1	**Yes**
10	10	10+ACE2	9.6	58.8	49.2	**No**	10	ACE2	10+ACE2	52.1	64.1	12	**No**
11	11	11+ACE2	21.8	80.7	58.9	**No**	11	ACE2	11+ACE2	52.3	83.6	31.3	**No**
12	14	14+ACE2	26.9	70.4	43.5	**+/-**	13	ACE2	14+ACE2	52.3	75.9	23.6	**No**
13	15	15+ACE2	10.1	50.7	40.6	**+/-**	14	ACE2	15+ACE2	50	59.7	9.7	**No**
14	17	17+ACE2	16.7	69.5	52.8	**No**	16	ACE2	17+ACE2	52	71.7	19.7	**No**
15	18	18+ACE2	12.6	52.5	39.9	**+/-**	17	ACE2	18+ACE2	50.9	61.7	10.8	**+/-**
16	20	20+ACE2	27.1	60.5	33.4	**+/-**	18	ACE2	20+ACE2	51.3	66.2	14.9	**+/-**
17	SR01	SR01+ACE2	39.4	92	52.6	**No**	19	ACE2	SR01+ACE2	51.4	97.2	45.8	**No**
18	SR02	SR02+ACE2	10.3	60.2	49.9	**No**	20	ACE2	SR02+ACE2	49.1	67.8	18.7	**No**
19	SR03	SR03+ACE2	18.5	70.7	52.2	**No**	21	ACE2	SR03+ACE2	50	76.4	26.4	**No**
20	SR04	SR04+ACE2	10.9	63.6	52.7	**No**	22	ACE2	SR04+ACE2	50.5	68.5	18	**No**
21	SR13	SR13+ACE2	36.9	86.8	49.9	**No**	12	ACE2	SR13+ACE2	52.3	92.8	40.5	**No**
22	SR16	SR16+ACE2	37.4	88.8	51.4	**No**	15	ACE2	SR16+ACE2	52.1	95.7	43.6	**No**
23	S2A3	S2A3+ACE2	15	82	67	**No**	23	ACE2	S2A3+ACE2	50.8	72.2	21.4	**No**
24	S2A4	S2A4+ACE2	11.6	73.3	61.7	**No**	24	ACE2	S2A4+ACE2	50.9	71.1	20.2	**No**
25	S2F3	S2F3+ACE2	50	111.1	61.1	**No**	25	ACE2	S2F3+ACE2	50.7	106.9	56.2	**No**
26	S2G3	S2G3+ACE2	59	113.2	54.2	**No**	26	ACE2	S2G3+ACE2	51	114.1	63.1	**No**

27	S2G4	S2G4+ACE2	21.3	81.7	60.4	**No**	27	ACE2	S2G4+ACE2	50.9	79.3	28.4	**No**
28	MRed04	MRed04+ACE2	31.9	73.3	41.1	**No**	29	ACE2	MRed04+ACE2	62.6	73.3	10.7	**+/-**
29	VHH-72	VHH-72+ACE2	25.8	37.8	12	**Yes**	28	ACE2	VHH-72+ACE2	51	51.2	0.2	**Yes**

[a]inj, injection. V_HHs were used at 20 – 40× K_D concentrations, ACE2 (ACE2-H_6) used at 1 μM.

Supplementary Table 8. Flow cytometry SVNAs against SARS-CoV-2 variants and SARS-CoV.

V_HH-Fc	SVNA IC_{50} (nM) SARS-CoV-2 S							SARS-CoV S
	Wuhan	Alpha	Beta	Gamma	Delta	Kappa	Omicron[a]	
**RBD-specific V_HH**								
1d	4.7	6.1	13.1	4.8	6.4	5.5	-	-
02	4.7	4.2	-	-	8.4	7.2	-	-
03	-	-	-	-	-	-	-	-
04	10.8	21.9	-	-	-	-	18.4	-
05	4.9	4.8	-	-	7.6	6.8	-	-
06	-	-	-	-	-	-	-	-
07	4.7	5.7	3.6	3.2	2.3	3.6	-	4.2
10	8.8	11.3	10.8	21.8	-	-	4.3	-
11	3.2	6.6	10.7	4.7	7.7	3.3	8.8	7.7
12	3.5	5.2	8	3.4	6.4	6.2	17.2	3.1
14	7.5	18	58	177	-	-	5.2	-
15	5.8	9.8	12.2	10.8	-	-	3	-
17	8.6	10.6	26.4	214	-	-	3.4	-
18	9.1	12.2	16.6	12.1	10.2	17.4	-	12.7
20	6.5	5.2	11.9	7.5	12.6	4.1	13.1	10.3
MRed04	5	6.4	24.4	8.7	11.8	10.4	-	24.6
MRed05	4.3	4.2	4.4	4.7	4.8	4.8	-	-
**NTD-specific V_HH**								
SR01	4.2	3.1	8.8	2.1	3.1	2.3	4.4	5.1
SR02	1.7	7.3	-	4.7	6.1	3.1	-	-
SR13	7.7	22.4	-	16.5	-	12.2	-	15
**Reference**								
VHH-72	5.6	10.6	5.1	3.3	10.5	8.5	-	7.8

[a]Omicron B.1.1.529. Dash indicates lack of neutralization.

Supplementary Table 9. Summary of HDX-MS experimental conditions and statistics.

Data Set	Batch 1	Batch 2	Batch 3	Batch 4	Batch 5	Batch 6	Batch 7
**Target protein**	RBD[b]	S1					S
**Protein states included**	VHH-72	03, 11, SR03, SR04, MRed03, MRed07	04, 05, 06, 10,14, 15	02, 17, 18, 1d, MRed04	07, 20, MRed05 SR01, SR02	12, MRed06, SR13, SR16	S2A3
**Technical replicates**	3						4
**HDX time course (min)**	5	3					1
**HDX reaction details**	10 mM Tris, pD = 7.0, 20°C in 45% D_2O						
**Protease for on-line digestion**	Pepsin						Nepenthesin-II
**LC gradient**	1-35% ACN, 8 min						2-40% ACN, 15 min
**# of peptides found across all samples**	69	215	222	219	196	217	278
**Sequence coverage**	76	71	75	75	75	74	75
**Average peptide length / Redundancy**	15.2/5.5	15.1/4.9	13.7/4.5	13.7 / 4.5	13.7/4.5	14.3/4.7	12.2 / 2.8
**Repeatability (average standard deviation)**	0.5	0.8	0.9	0.9	0.9	0.7	1.2
**3 x SD cut-off[a]**	4.3	6	4.7	5	4.9	6.3	3.5
**Significant differences in HDX**	Two-state student T-Test perfomed for each state (ΔD > 3 x SD, 1-p value > 0.98)						

[a]SD cutoff was calculated for each state within a batch, and the highest cutoff was applied to all states in that batch; [b]RBD_short (aa331-521).

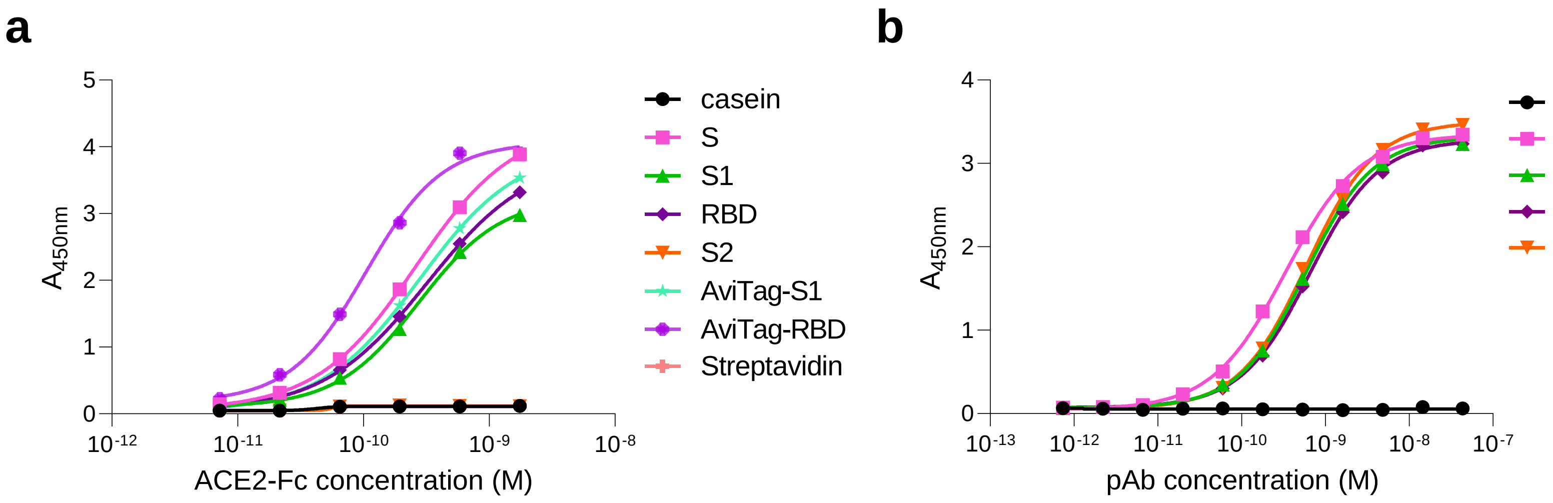

Antigen	S	S1	S2	RBD	AviTag-RBD	AviTag-S1
EC_{50} (nM)	0.26	0.28	-	0.32	0.1	0.28

Antigen	S	S1	S2	RBD
EC_{50} (nM)	0.34	0.59	0.6	0.65

Supplementary Figure 1. Antigen validation. Prior to immunization, serology and panning experiments, SARS-CoV-2 spike protein antigens were validated for functionality in adsorbed/captured states on microtiter wells by measuring their binding to ACE2 and an anti-S polyclonal antibody. **a** ELISA assessing the binding of microtiter well-adsorbed (S, S1, S2 and RBD) and microtiter well-captured (AviTag-S1, AviTag-RBD) SARS-CoV-2 spike glycoprotein fragments to their human receptor ACE2 (fused to human IgG1, ACE2-Fc). AviTag-S1 and AviTag-RBD were captured on streptavidin-coated microtiter wells through their C-terminal biotin tag. **b** ELISA confirming the binding of microtiter well-adsorbed SARS-CoV-2 spike glycoprotein fragments S, S1, S2 and RBD to a commercial rabbit anti-SARS-CoV-2 S polyclonal antibody (pAb). No binding was observed to S2 (**a**) or to casein (**a** & **b**), demonstrating binding specificities. Calculated EC_{50}s (half-maximal effective concentration) are tabulated below the graphs. SARS-CoV-2 Wuhan spike glycoprotein fragments were used in the assays.

a

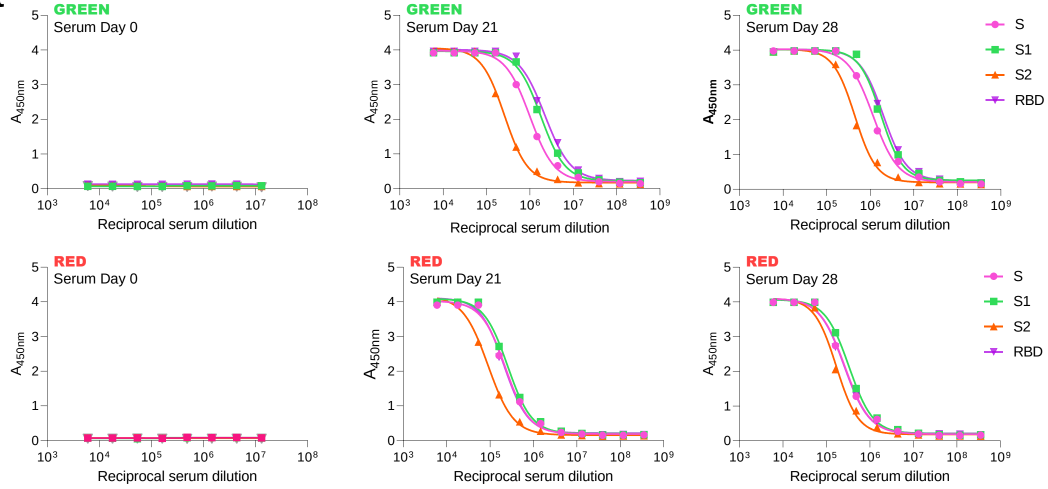

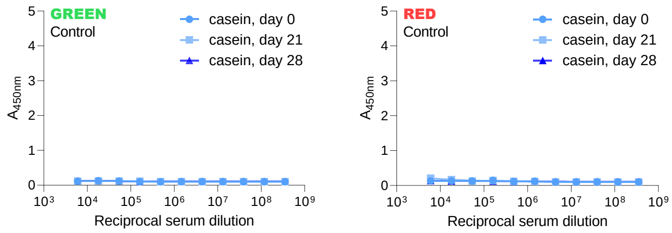

Llama	Day	*EC*$_{50}$ (×10^{6} recirocal serum dilution)			
		S	S1	S2	RBD
Green	0	-	-	-	-
	21	1	1.8	0.3	2.2
	28	1.2	1.8	0.4	2
Red	0	-	-	-	-
	21	0.2	0.3	0.1	0.2
	28	0.3	0.3	0.2	0.3

b

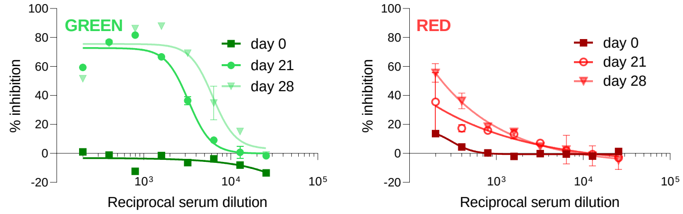

Llama	*IC*$_{50}$ (recirocal serum dilution)	
	Day 21	Day 28
Green	3300	6200
Red	<200	<400

Supplementary Figure 2. Llama serology. a ELISA performed with pre-immune (day 0) and immune (days 21 and 28) sera demonstrated SARS-CoV-2 spike glycoprotein-immunized llamas Green and Red generated strong immune responses towards S, S1, S2 and RBD. ELISA performed with day 0, 21 and 28 sera showed immunized llama sera did not react with non-target antigen (casein), demonstrating specificity of the immune response. EC_{50}, measures of the strength of immune response, are shown in the table. Based on EC_{50} values, Green generated a stronger immune response in comparison to Red, up to 10-fold, consistently across all four spike protein fragments. **b** Flow cytometry surrogate virus neutralization assays (SVNAs) performed with pre-immune (day 0) and immune (days 21 and 28) sera demonstrated llama Green mounted a polyclonal immune response that was more potent in blocking the binding of SARS-CoV-2 S to Vero E6 cell surface-associated ACE2 in comparison to llama Red. IC_{50}s (half-maximal inhibitory concentrations), measures of the strength of serum neutralization capability, are shown in the table. Due to a lack of complete curves, IC_{50}s for llama Red sera were estimated by assuming similar upper plateaus as those for

llama Green sera. IC_{50}s of 3300 (day 21) and 6200 (day 28) reciprocal serum dilution (RSD) were obtained in the case of the Green sera compared to far weaker ones, <200 RSD (day 21) and <400 RSD (day 28), for the Red sera. SARS-CoV-2 Wuhan spike glycoprotein fragments were used for immunizations and in assays. Error bars indicate standard deviation (SD) of two technical replicates.

a

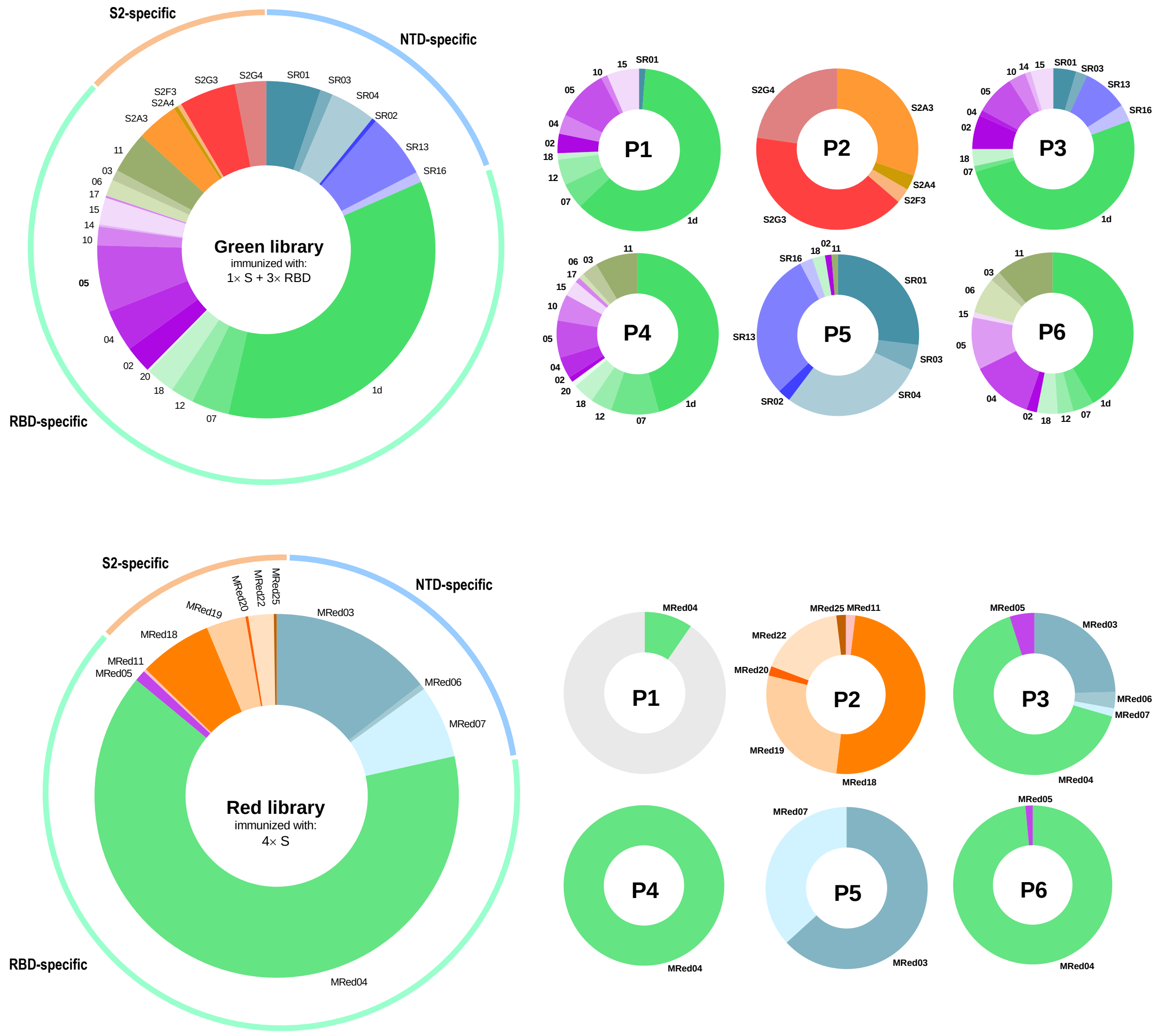
S2-specific
NTD-specific
RBD-specific
Green library
immunized with:
1× S + 3× RBD
P1
P2
P3
P4
P5
P6
Red library
immunized with:
4× S
MRed03
MRed04
MRed05
MRed06
MRed07
MRed11
MRed18
MRed19
MRed20
MRed22
MRed25

b

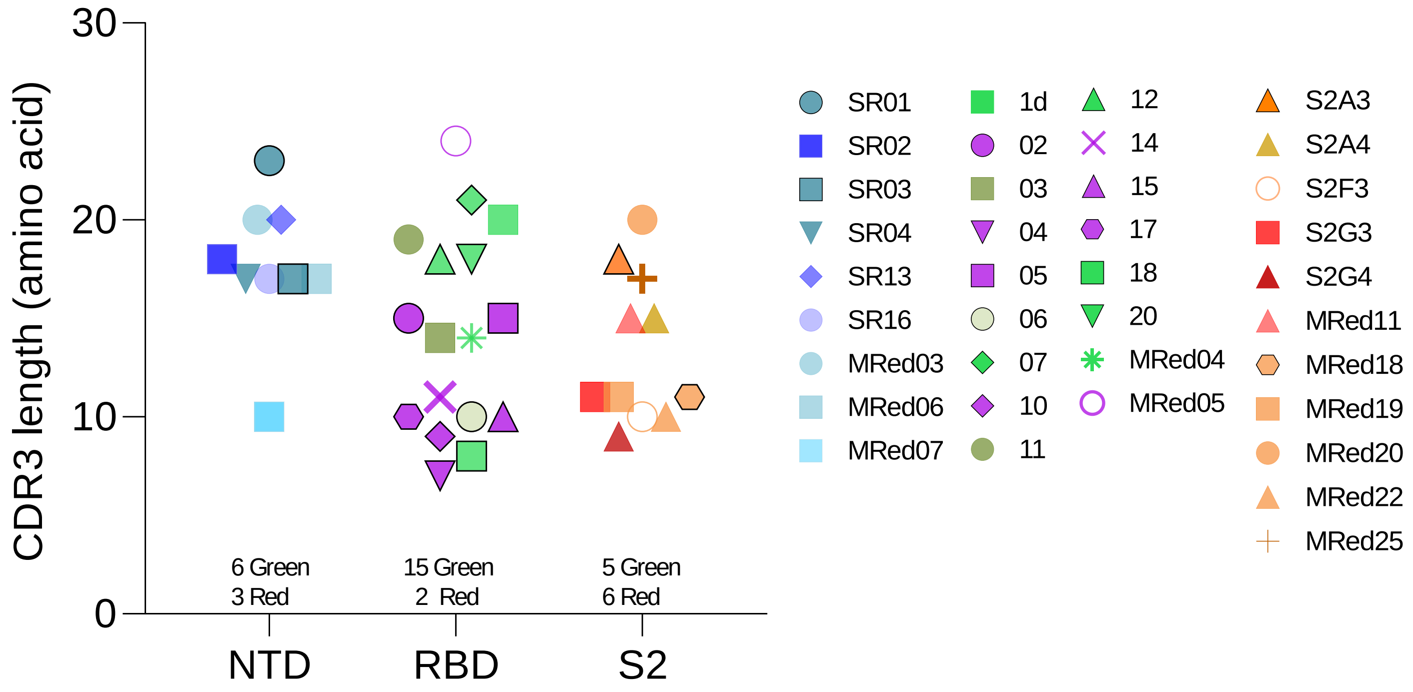

Supplementary Figure 3. Selection, binding, subunit/domain specificity and CDR3 length heterogeneity of anti-SARS-CoV-2 S V_HHs. V_HHs are grouped based on their specificity for NTD, RBD or S2 and color-coded based on their epitope bin designation (*see* Fig. 1d). **a** Library origin, relative proportion and subunit/domain specificity of the 37 anti-SARS-CoV-2 S V_HHs selected from the Green and Red libraries by employing six panning strategies P1 – P6. Binding specificity patterns in P1 – P6 reflect the selection strategies employed. For the Red library, P1 also selected for a V_HH (MRed09, grey, unlabeled) that was specific to the resistin trimerization domain of the S used for immunization and panning. Green library V_HHs were isolated from the llama immunized once with S and three times with RBD. Red library V_HHs were isolated from the llama immunized four times with S. **b** Diversity of the 37 SARS-CoV-2 S V_HHs shown in terms of CDR3 length. The number of V_HHs derived from each of the two libraries are shown.

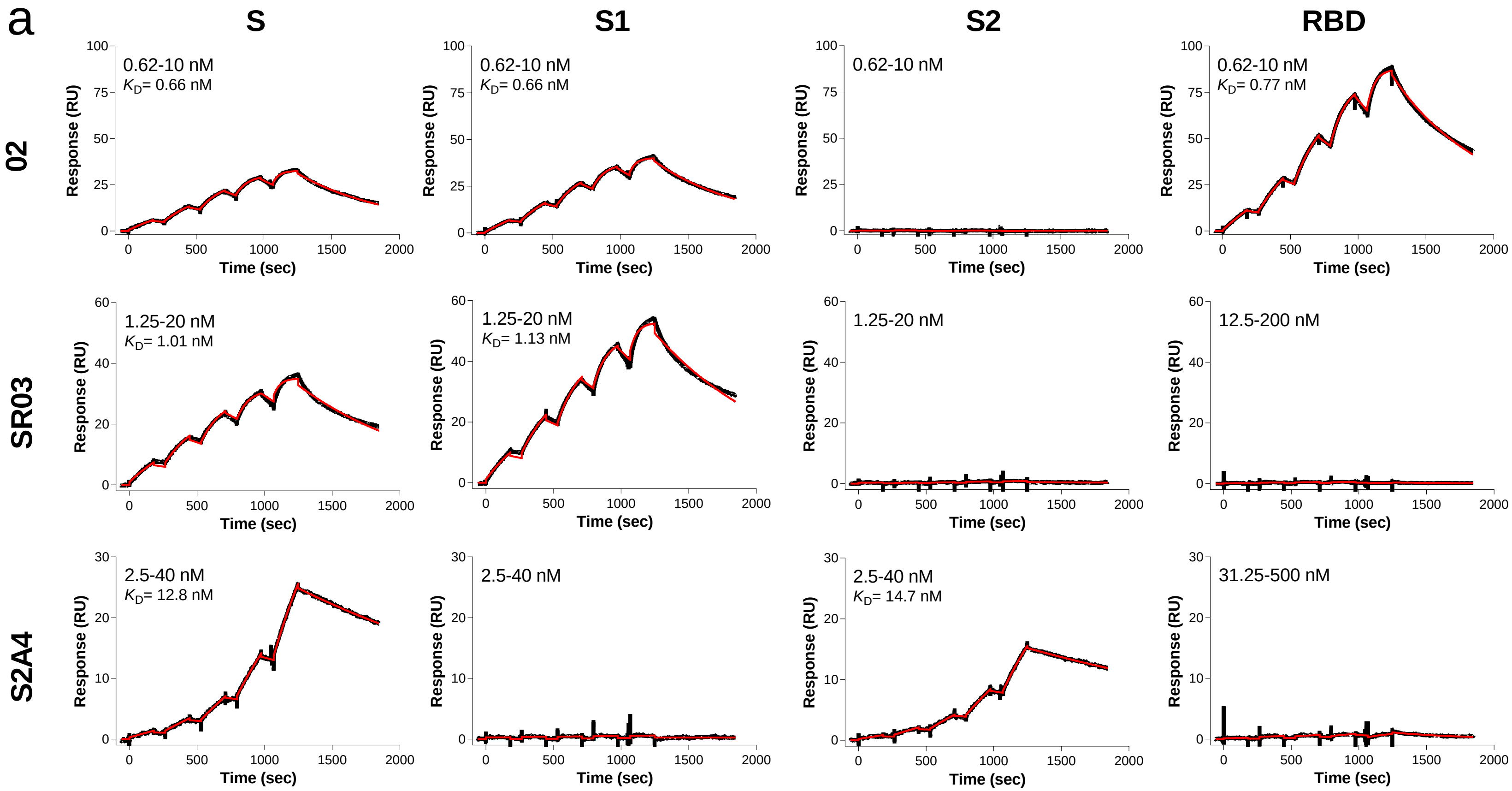
a
S
S1
S2
RBD
02
SR03
S2A4
0.62-10 nM
K_D= 0.66 nM
0.62-10 nM
K_D= 0.66 nM
0.62-10 nM
0.62-10 nM
K_D= 0.77 nM
1.25-20 nM
K_D= 1.01 nM
1.25-20 nM
K_D= 1.13 nM
1.25-20 nM
12.5-200 nM
2.5-40 nM
K_D= 12.8 nM
2.5-40 nM
2.5-40 nM
K_D= 14.7 nM
31.25-500 nM
Response (RU)
Time (sec)

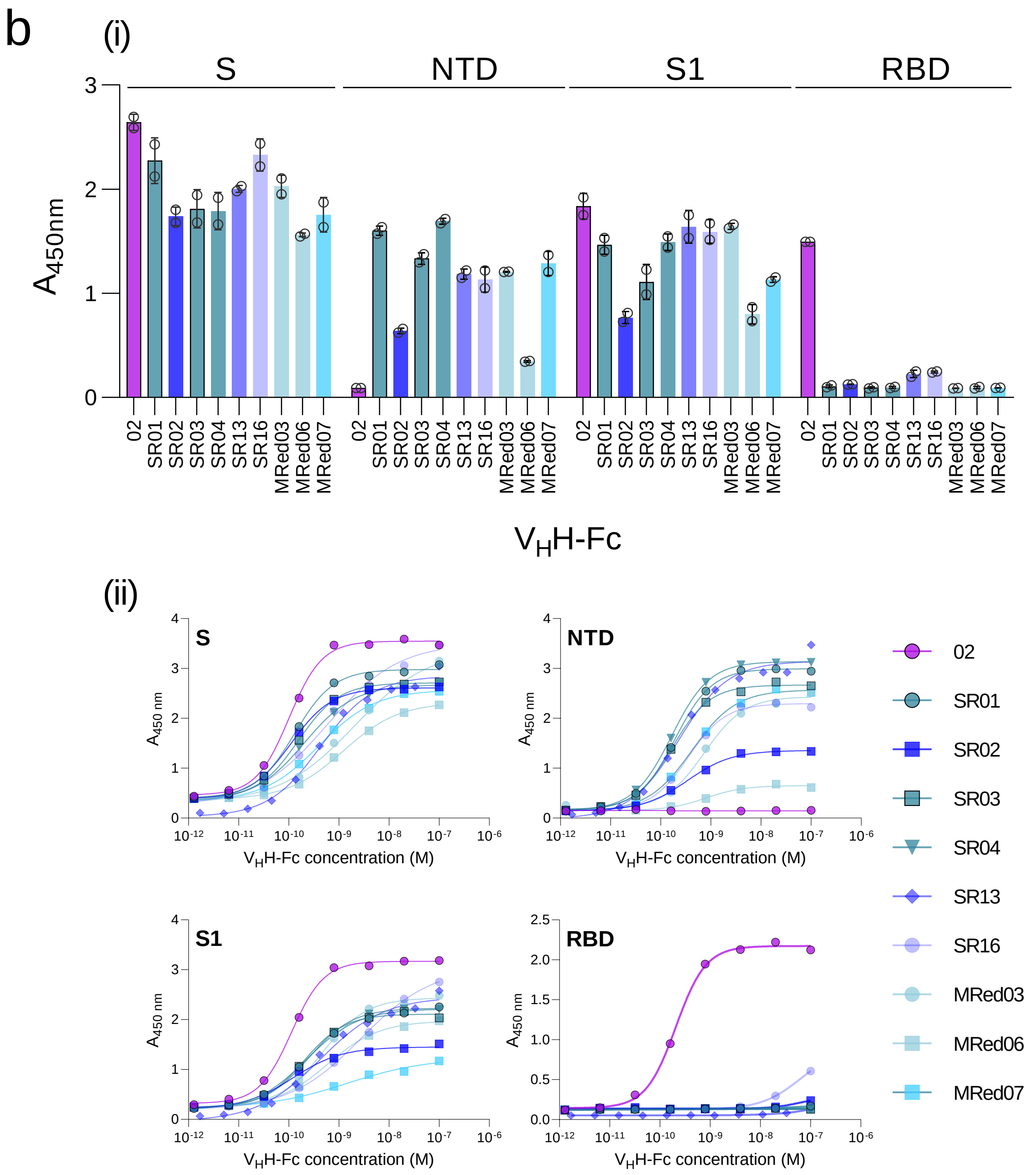

Supplementary Figure 4. Binding affinity and subunit/domain specificity of SARS-CoV-2 V$_H$Hs and V$_H$H-Fcs by SPR and ELISA. a Representative SPR sensorgrams showing single-cycle kinetics analysis of 02, SR03 and S2A4 V$_H$Hs binding to SARS-CoV-2 S, S1, S2 and RBD. Spike glycoprotein fragments were immobilized on CM5 sensorchip surfaces and V$_H$Hs flowed over at concentration ranges described in the figure. Black lines represent raw data points; red lines are fits to a 1:1 binding model. 02, SR03 and S2A4 antibodies represent SPR binding profiles for V$_H$Hs specific to RBD, NTD and S2, respectively. Calculated kinetic and equilibrium dissociation constants (k_as, k_ds and K_Ds) obtained for binding of V$_H$Hs against SARS-CoV-2 spike glycoprotein fragments are reported in Table 1, Supplementary Table 2 and Fig. 1b. **b** ELISA

assessing the domain specificity of SARS-CoV-2 V_HH-Fcs. Assays were performed against SARS-CoV-2 S, S1, NTD and RBD at a fixed (13 nM) (**i**) or varying (**ii**) concentrations of V_HH-Fcs. 02 is included as internal control. Error bars indicate standard deviation (SD) of two technical replicates (**i**)**.** (**ii**) Apparent EC_{50}s (EC_{50}apps) obtained from graphs are recorded in Supplementary Table 3. SARS-CoV-2 Wuhan spike glycoprotein fragments were used in assays.

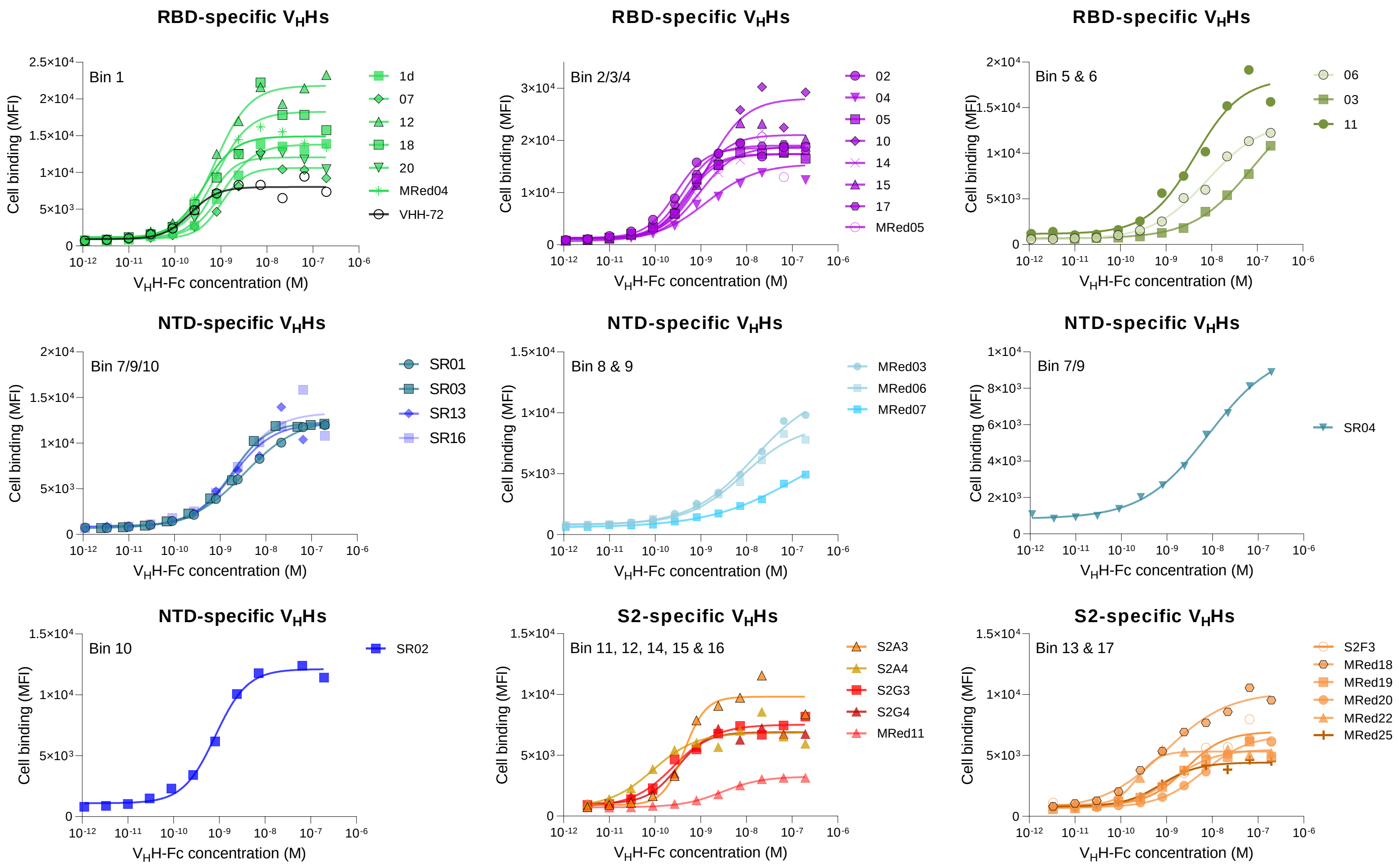

Supplementary Figure 5. Assessing binding of SARS-CoV-2 VHH-Fcs to S-expressing CHO cells (CHO-SPK) by flow cytometry. Calculated apparent EC_{50}s (EC_{50}apps) obtained from graphs are reported in Table 1 and used in Fig. 1c. None of the VHH-Fcs bound to parental non-S-expressing CHO cells at the highest concentration tested (200 nM). MFI, mean fluorescence intensity.

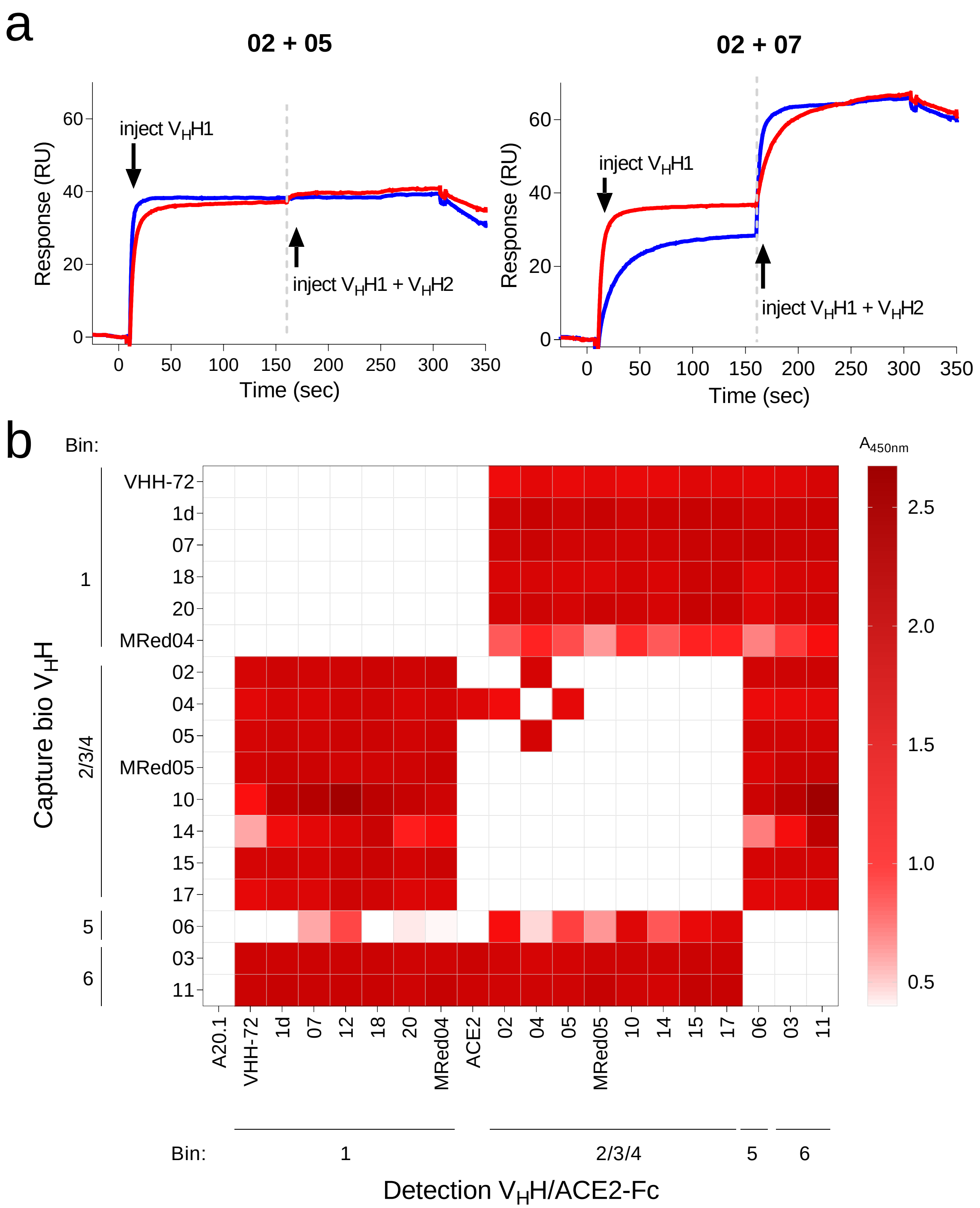

Supplementary Figure 6. Epitope binning of SARS-CoV-2 VHHs. a Epitope binning by SPR co-injections. Representative sensorgrams showing SPR epitope binning on SARS-CoV-2 Wuhan S-immobilized surface. For each test pair (02 + 05 or 02 + 07), the first VHH (VHH1) was injected over immobilized S at saturating antigen binding concentration (50× K_D) followed by a second injection containing a mixture of the first

V_HH and a second V_HH (V_HH1 + V_HH2), both at saturating concentrations (50× K_D). Assays were performed in two orientation formats: injecting V_HH1 first (orientation #1; blue profile) or V_HH2 first (orientation #2; red profile), followed by injecting V_HH1 + V_HH2 mix. The 02 + 05 profile represents V_HH pairs that are interpreted to bind to overlapping epitopes, hence belonging to the same epitope bin, since the injection of the second V_HH does not result in significant increase in binding (RU) over that already achieved by the injection of the first V_HH. Conversely, the 02 + 07 profile represents V_HH pairs that are interpreted to bind to non-overlapping epitopes and belong to different epitope bins, since the addition of the second V_HH results in a significant increase in binding over that achieved by the injection of the first V_HH. **b** Epitope binning of RBD V_HHs by competitive sandwich ELISA. Biotinylated V_HHs (bio V_HHs) were captured on streptavidin-coated wells, followed by the addition of SARS-CoV-2 Wuhan S1. The second, detecting V_HH sets were added as bivalent V_HH-Fcs, followed by the addition of HRP-conjugated anti-Fc antibody to probe V_HH-Fc binding to S1. ELISA binding results for pair-wise combinations of V_HHs against S1 are presented as a heat map. Binding pairs giving a binding signal (color) were considered as recognizing non-overlapping epitopes and belong to different epitope bins, while those giving no/weak binding signals (colorless/pale color) were considered recognizing overlapping epitopes and belong to the same epitope bin. V_HH 12 is not included on the vertical axis as it could not be produced in biotinylated form.

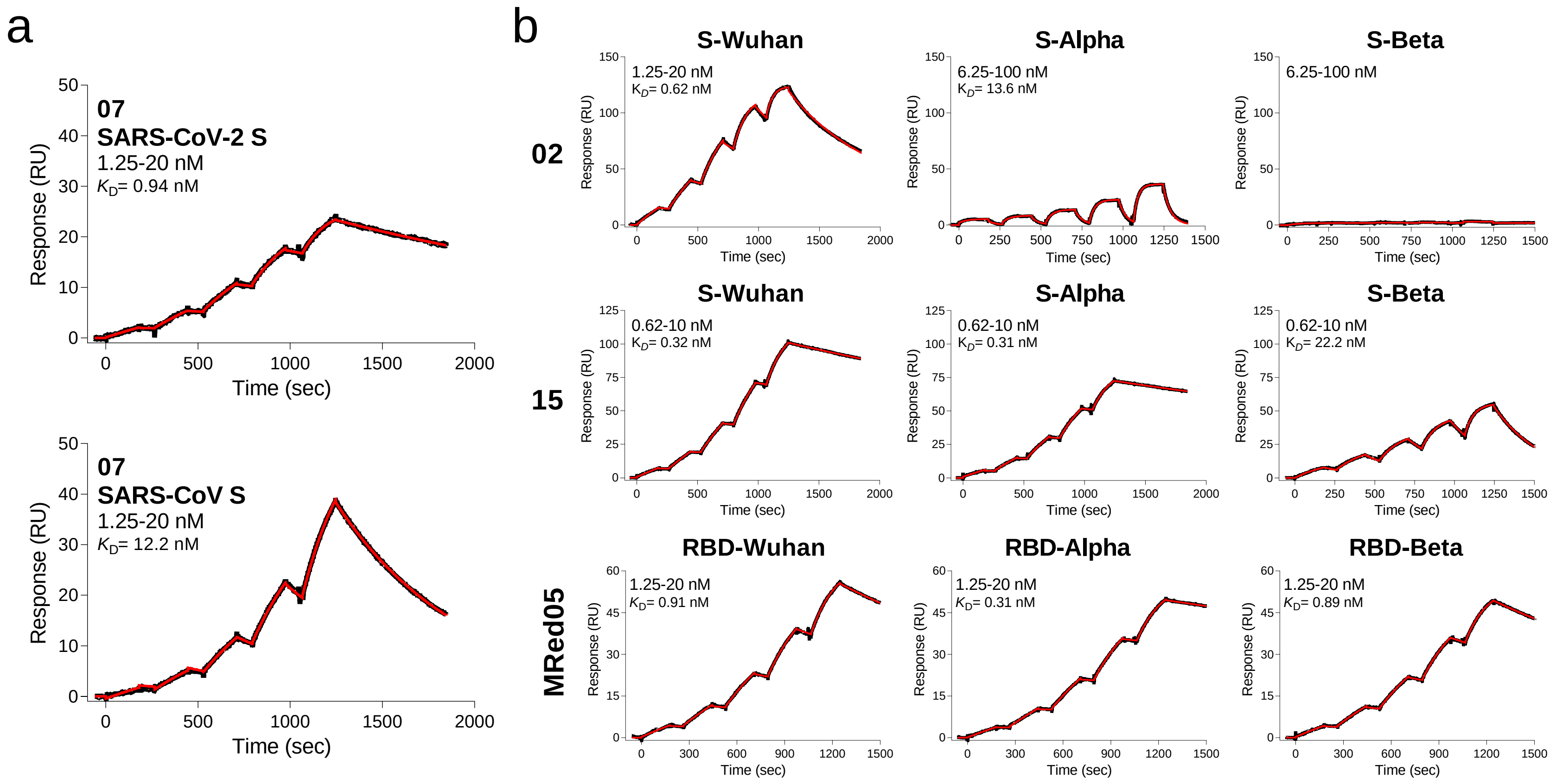

Supplementary Figure 7. Cross-reactivity assessment of SARS-CoV-2 V_HHs by SPR. Representative SPR sensorgrams showing single-cycle kinetics analysis of 07 binding to SARS-CoV-2 S and SARS-CoV S (**a**), and 02, 15 and MRed05 binding to Wuhan, Alpha and Beta S (02, 15) and RBD (MRed05) (**b**). Spike glycoproteins (S) were immobilized on CM5 sensorchip surfaces followed by flowing 07, 02 and 15 V_HHs at concentration ranges described in the figure. MRed05 V_HH-Fc was captured on anti-human Fc sensorchip surfaces followed by flowing RBDs at concentration ranges described in the figure. Black lines represent raw data points; red lines are 1:1 model fits. Calculated kinetic and equilibrium dissociation constants (k_as, k_ds and K_Ds) obtained for binding of V_HHs/V_HH-Fc to spike glycoprotein fragments are reported in Fig. 2b, Fig. 2c, Table 1 and Supplementary Table 4.

RBD-specific V_HHs

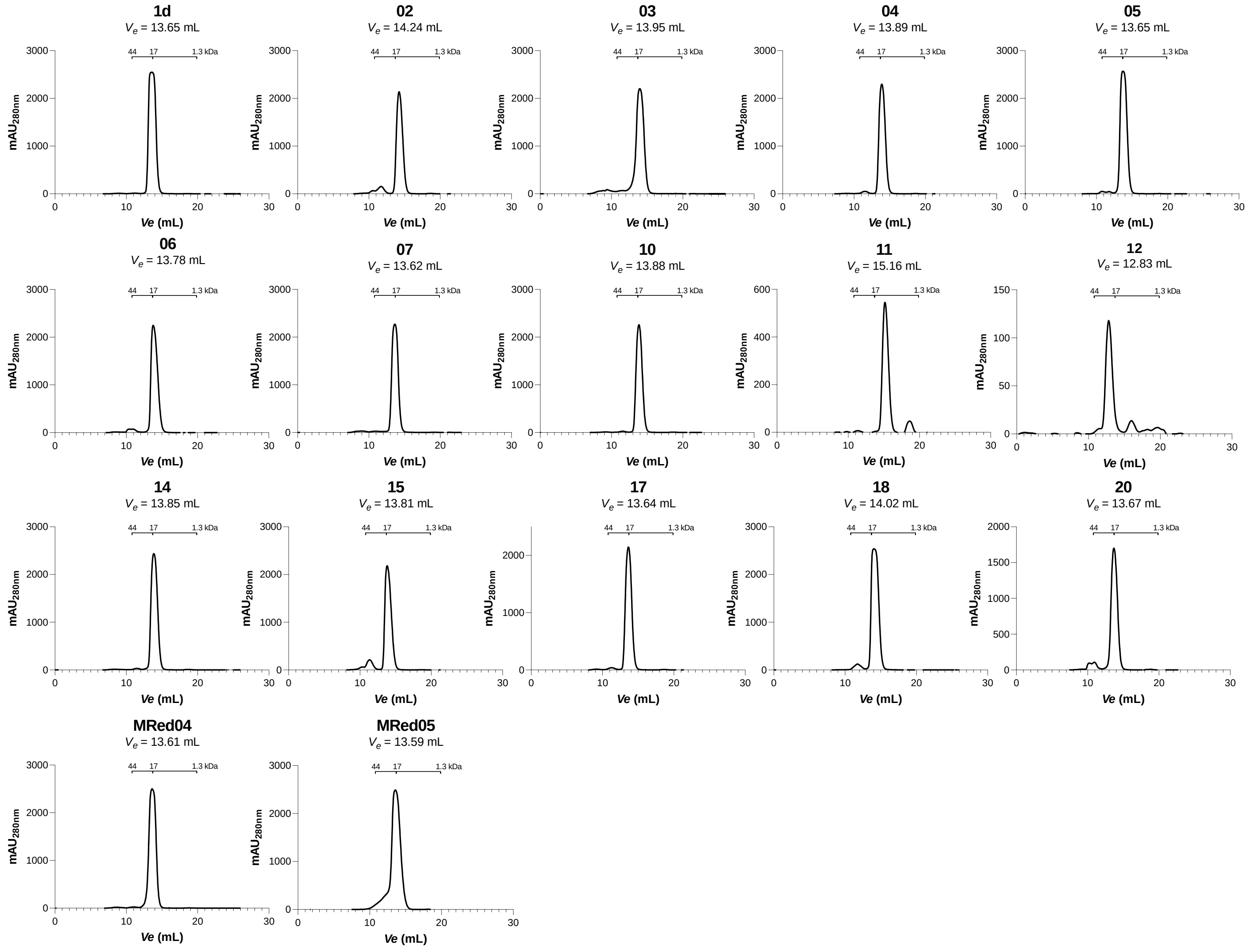

NTD-specific V_HHs

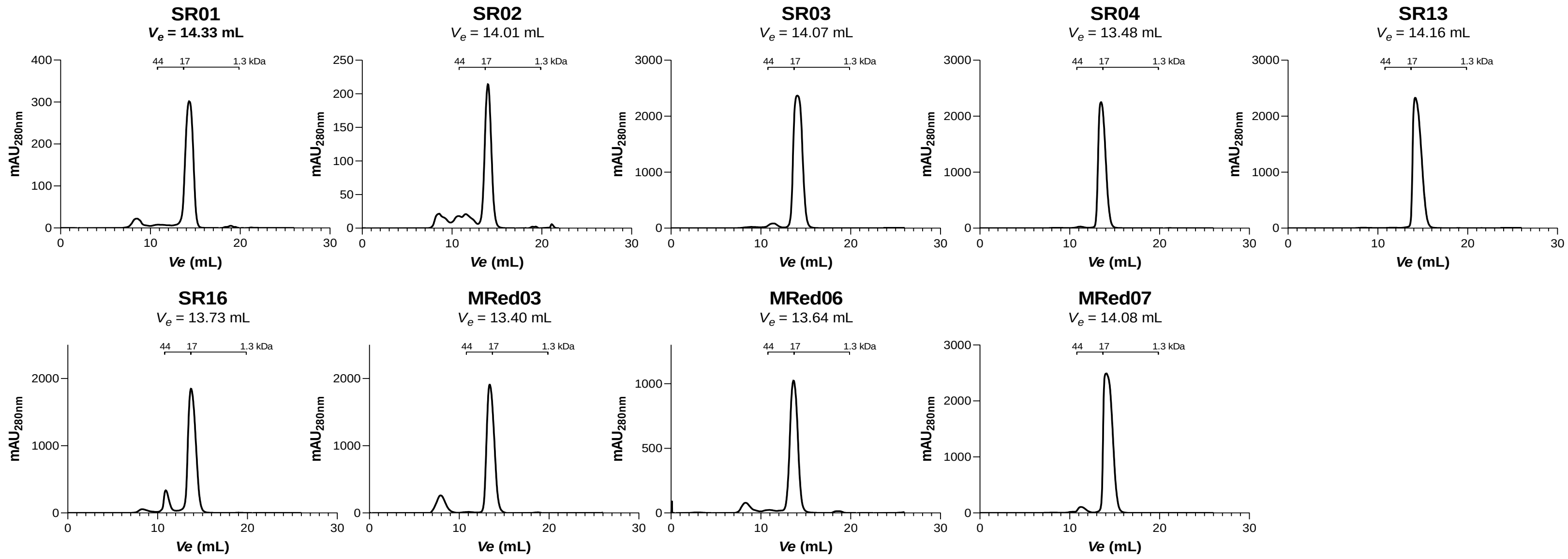

S2-specific V_HHs

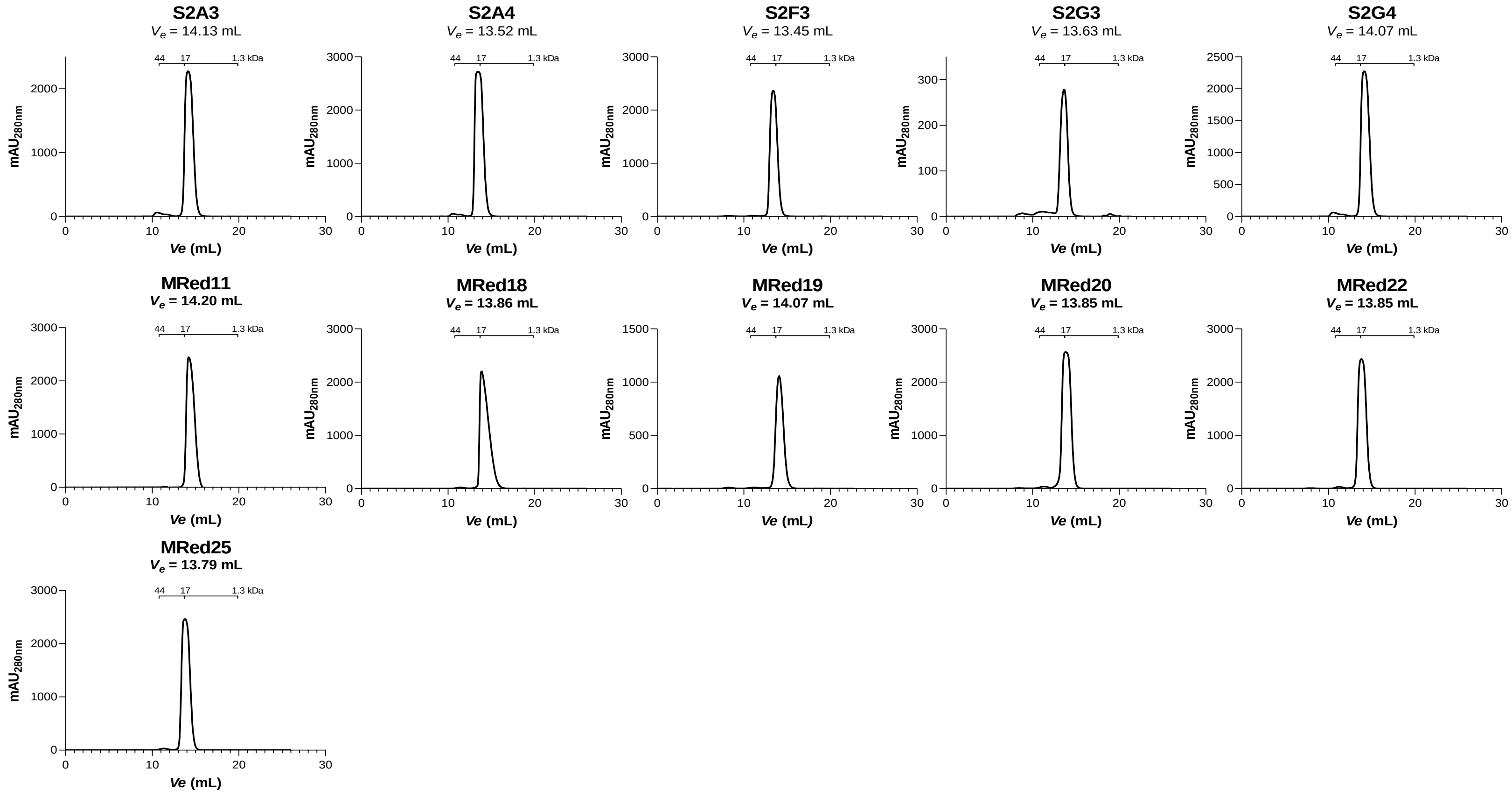

Supplementary Figure 8. Aggregation propensity of SARS-CoV-2 V_HHs. SEC profiles of SARS-CoV-2 V_HHs demonstrated the V_HHs were aggregation free and had elution volumes (V_es) consistent with their monomeric states. The V_e positions of molecular mass standards (44 kDa, 17 kDa, 1.3 kDa) are shown. SEC was performed using a Superdex™ 75 Increase column. mAU, milliabsorbance unit.

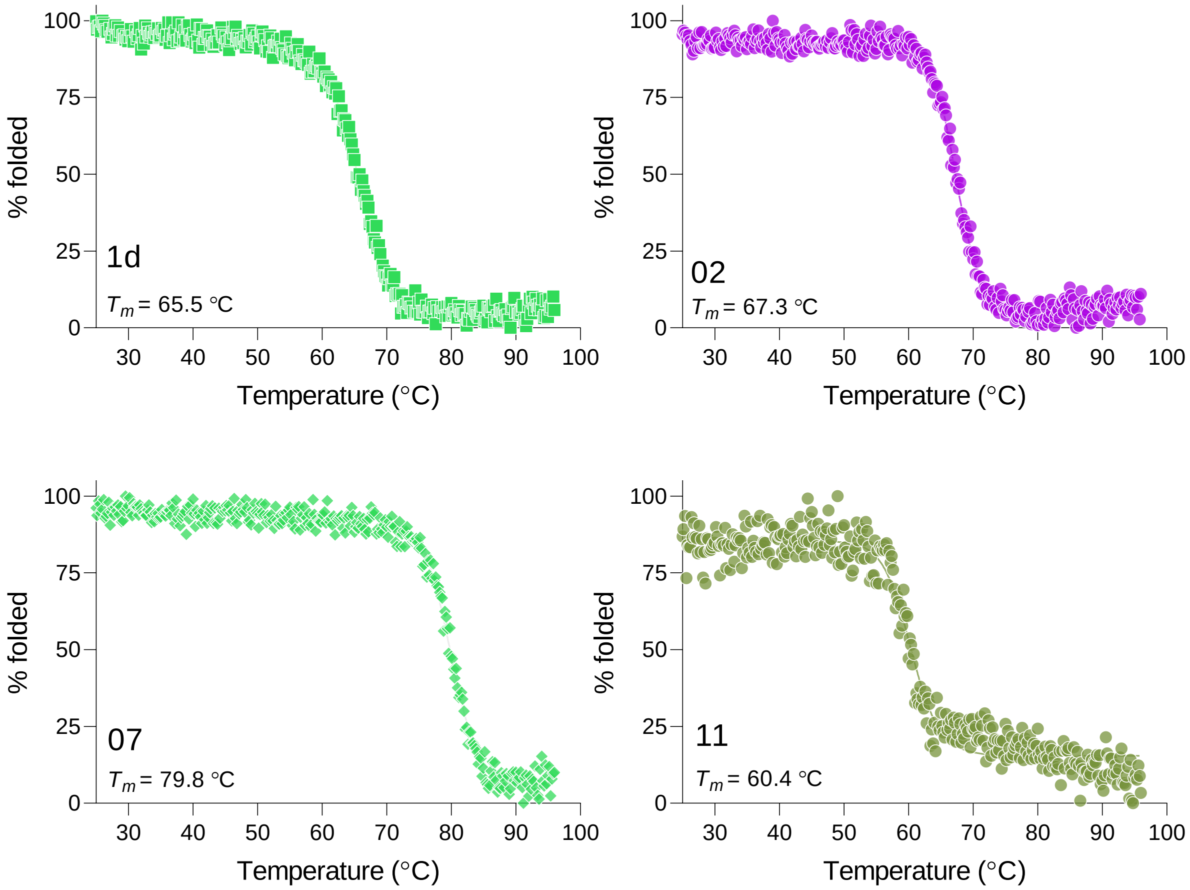

Supplementary Figure 9. Temperature-induced unfolding profiles of SARS-CoV-2 V_HHs. Representative examples showing the thermal unfolding of 1d, 02, 07 and 11 determined using circular dichroism (CD) spectroscopy. V_HH thermal unfolding midpoint temperatures (T_ms), obtained from graphs of % folded *vs* temperature, were used to construct Fig. 3b and Supplementary Table 5.

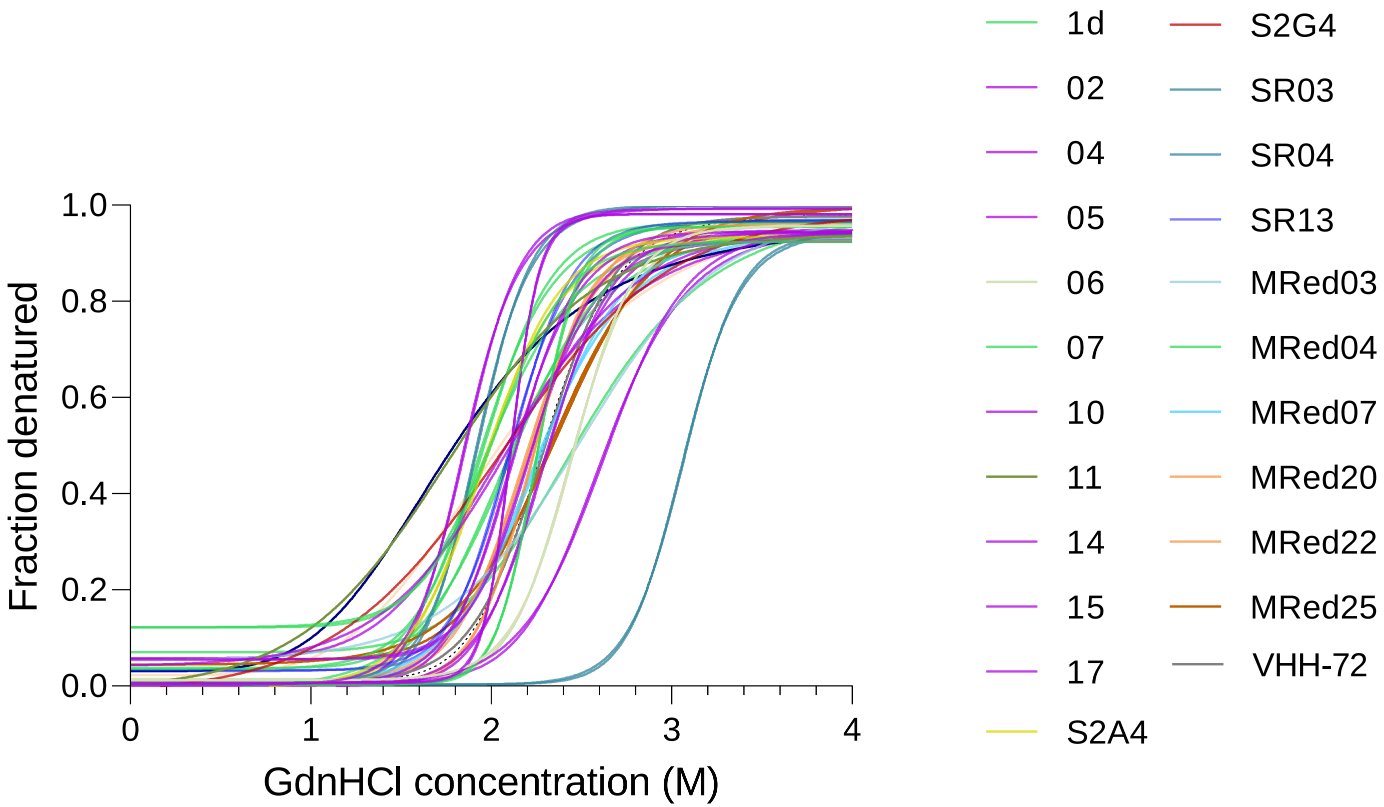

Supplementary Figure 10. GdnHCl-induced unfolding profiles of SARS-CoV-2 V_HHs. Unfolding of V_HHs was monitored using the automated Hunky system as described in Methods. Plots of fraction denatured *vs* denaturant (GdnHCl) concentration confirm the two-state transition model. The midpoints of the unfolding transition, C_ms, are reported in Supplementary Table 5. GdnHCl, guanidine-HCl.

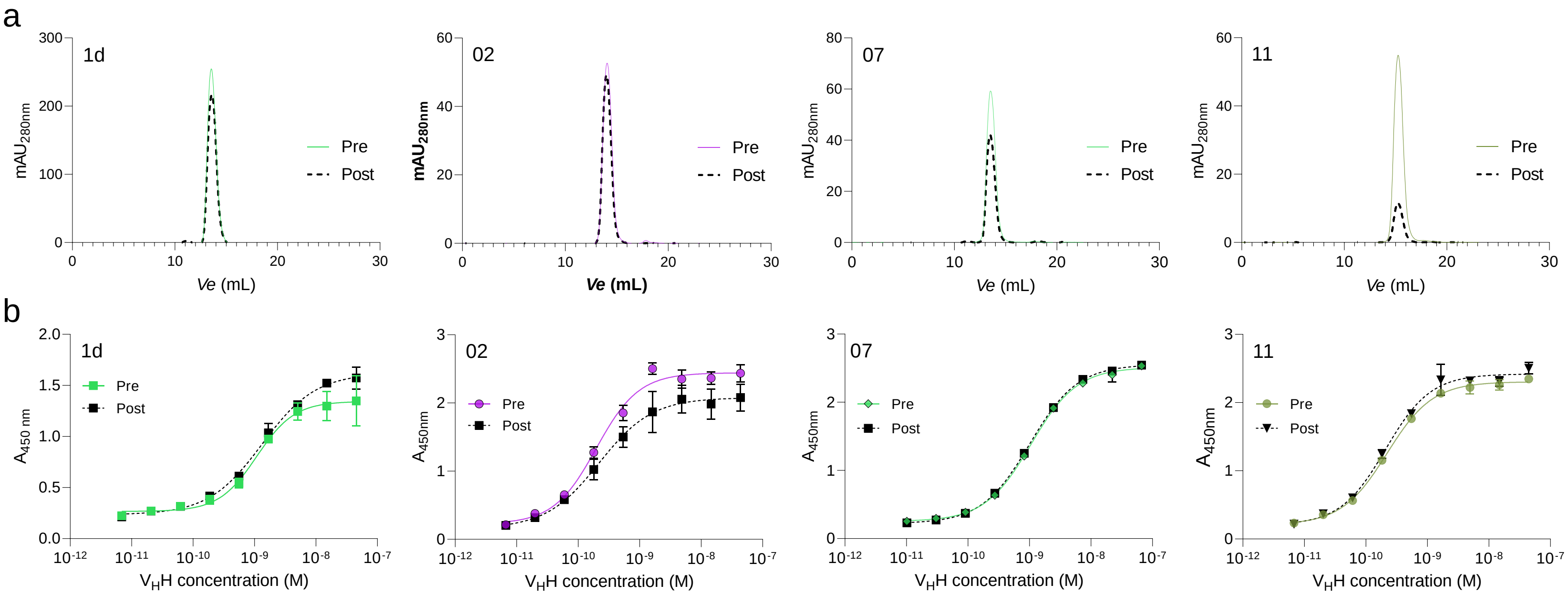

Supplementary Figure 11. Stability of SARS-CoV-2 V_HHs against aerosolization. a Assessing the effect of aerosolization on the aggregation resistance of V_HHs by comparing the SEC profiles of pre- *vs* post-aerosolized V_HHs. Results for representative V_HHs are shown. 1d, 02 and 07 represent the majority of V_HHs which were resistant to aerosolization-induced aggregation, showing homogenous monomeric peaks following aerosolization. V_HH 11 represents one of the few V_HHs which formed visible, precipitating aggregates reflected by a significant reduction of monomeric peak area (compare monomeric peak for pre- *vs* post-aerosolized V_HH 11). Aggregation data for a complete set of V_HHs are summarized in Supplementary Table 6, Fig. 3e (left panel) and Fig. 3e (middle panel). **b** ELISA assessing the effect of aerosolization on the functionality of V_HHs by comparing the binding activity of pre- *vs* post-aerosolized V_HHs against SARS-CoV-2 S. Essentially identical EC_{50}s for pre- *vs* post-aerosolized V_HHs indicates aerosolization had no effect on the functional activity of V_HHs. EC_{50}s are summarized in Fig. 3e (right panel). Pre, pre-aerosolized V_HH; post, post-aerosolized V_HH; mAU, milliabsorbance; V_e, elution volumes. Error bars indicate standard deviation (SD) of two technical replicates (**b**).

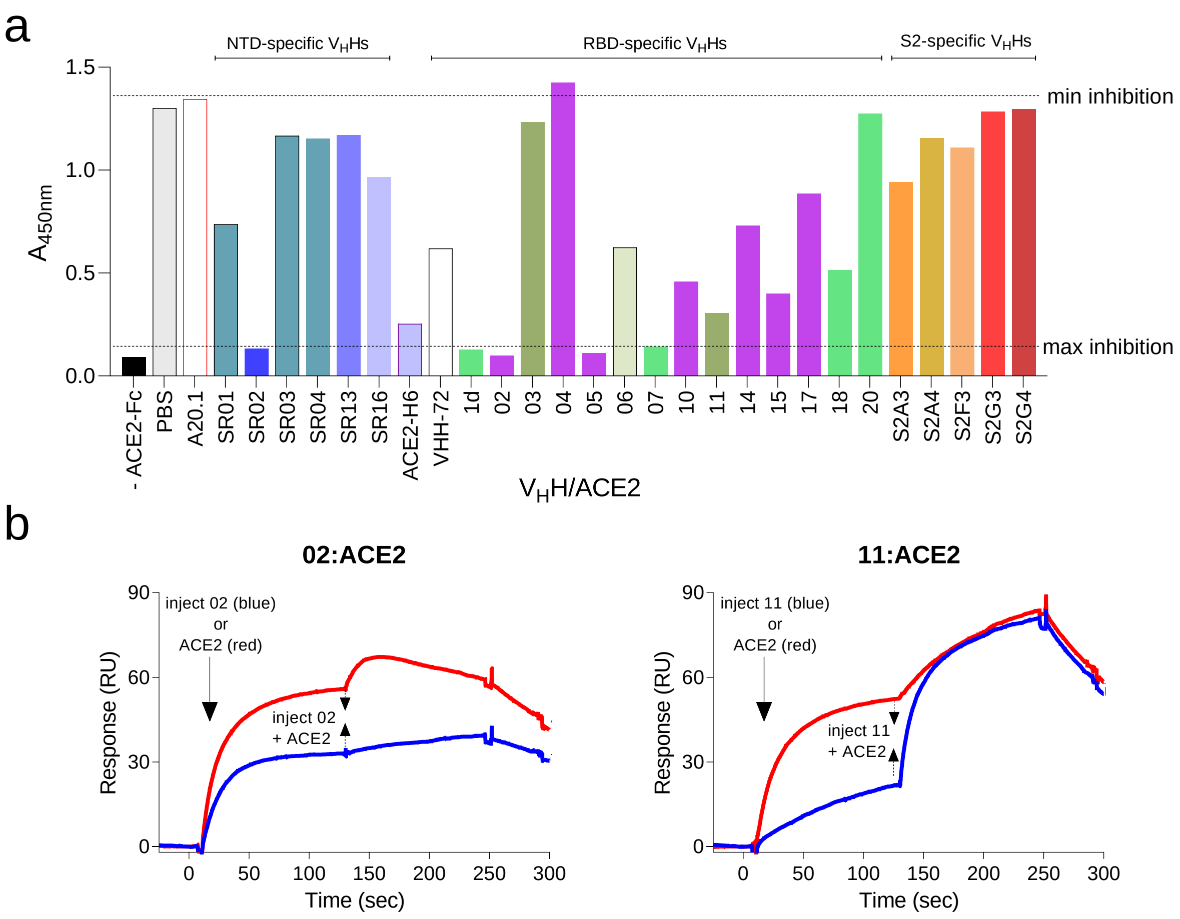

Supplementary Figure 12. V_HH SVNAs by ELISA and SPR. a ELISA assessing the ability of monomeric V_HHs to block the binding of ACE2 to SARS-CoV-2 S. A dimeric, Fc-fused ACE2 (ACE2-Fc) was used in the assays at 100 ng and mixed with a fixed concentration of V_HHs (1 µM). VHH-72 V_HH[7] and monomeric ACE2-H_6 served as positive and reference controls, while toxin A-specific A20.1 V_HH[9] was a negative control. "PBS" and "A20.1" represent assays in which a SARS-CoV-2 V_HH was substituted with PBS or A20.1 V_HH and provide reference binding signals for lack of blocking ("min inhibition"). "-ACE2-Fc" control represent assays with ACE2-Fc omitted and provides a reference binding signal for 100% blocking ("max inhibition"). V_HHs are color-coded according to their epitope bin designation (*see* Fig. 1d). **b** Representative sensorgrams assessing the ability of monomeric V_HHs in blocking the binding of ACE2 receptor to its ligand SARS-CoV-2 S. SPR assays were performed in two orientations, where injection of V_HH (orientation #1) or ACE2 (orientation #2) at 20 – 40× K_D concentration (V_HH) or 1 µM (ACE2) over immobilized S, was followed by injection of a mixture of V_HH + ACE2 at the same V_HH and ACE2 concentrations. Blue and red profiles represent binding results with the two orientations. "02:ACE2" represents a profile for a blocking V_HH, where the addition of the V_HH or ACE2 results in no significant increase in binding over that achieved by the injection of the ACE2 or V_HH on

the antigen surface. “11:ACE2” represents a profile for non-blocking V_HHs, where the addition of the V_HH or ACE2 results in significant increase in binding over that achieved by the injection of the ACE2 or V_HH on the antigen surface. ΔRUs, representing binding differences between the first and second injection, were calculated from the sensorgrams and used to identify V_HHs that block the binding of ACE2 receptor to its ligand RBD (*see* Supplementary Table 7). SARS-CoV-2 Wuhan S were used in assays. ACE2, monomeric ACE2-H_6.

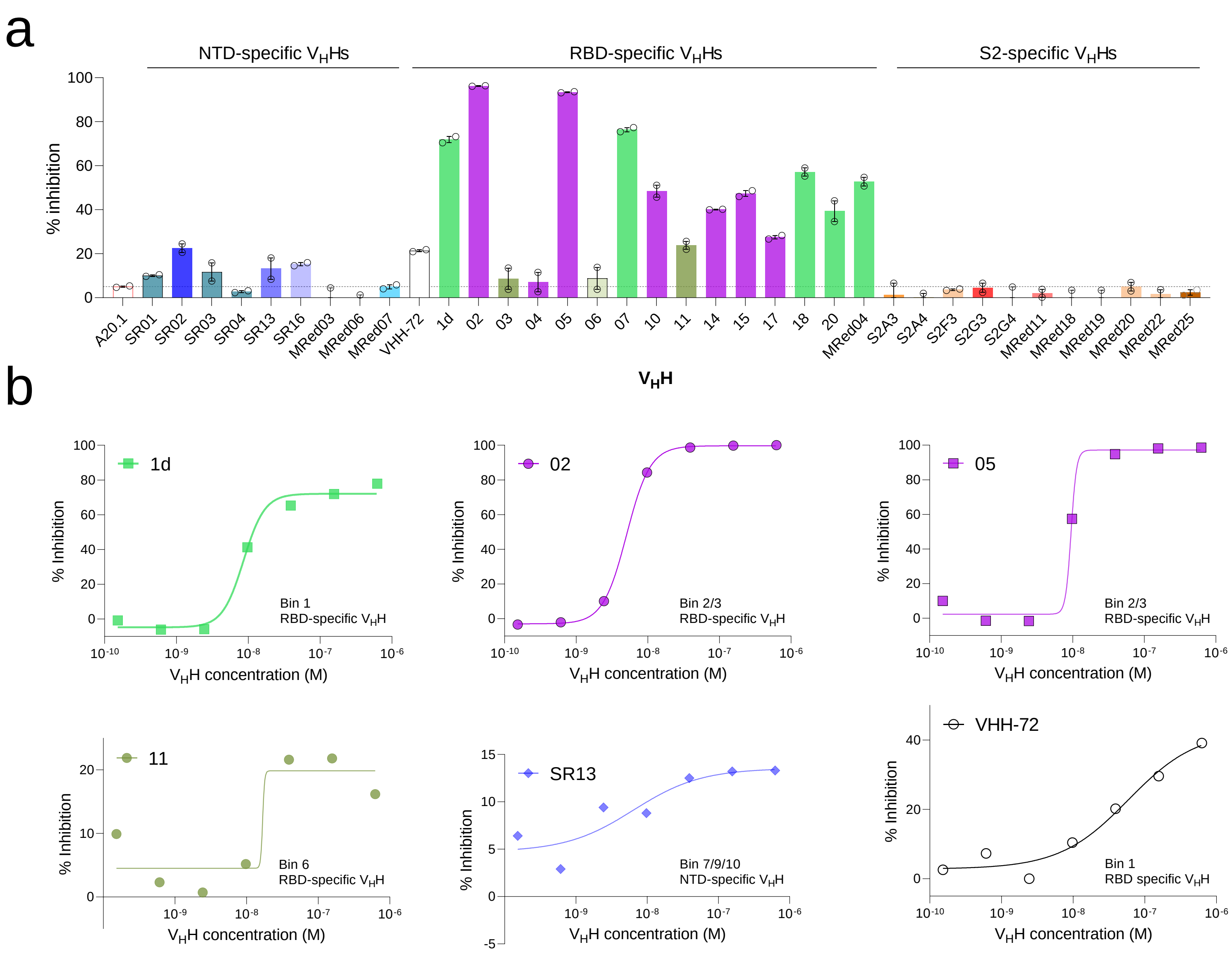
a
NTD-specific VHHs
RBD-specific VHHs
S2-specific VHHs
% inhibition
A20.1
SR01
SR02
SR03
SR04
SR13
SR16
MRed03
MRed06
MRed07
VHH-72
1d
02
03
04
05
06
07
10
11
14
15
17
18
20
MRed04
S2A3
S2A4
S2F3
S2G3
S2G4
MRed11
MRed18
MRed19
MRed20
MRed22
MRed25
VHH
b
1d
Bin 1
RBD-specific VHH
02
Bin 2/3
RBD-specific VHH
05
Bin 2/3
RBD-specific VHH
11
Bin 6
RBD-specific VHH
SR13
Bin 7/9/10
NTD-specific VHH
VHH-72
Bin 1
RBD specific VHH
% Inhibition
VHH concentration (M)

Supplementary Figure 13. Flow cytometry SVNAs assessing the ability of monomeric SARS-CoV-2 V_HHs in blocking the binding of SARS-CoV-2 S to ACE2-expressing Vero E6 cells at 100 nM (a) or varying (b) V_HH concentrations. Representative plots are shown in **b**. V_HHs 1d, 02, 05 and 11 are RBD-specific, SR13 is NTD-specific. The complete list of IC_{50}s calculated from graphs exemplified in **b** are shown in Table 2. Monomeric “ACE2” (ACE2-H_6) serves as positive “antibody” control and reference; VHH-72 V_HH[7] is included as benchmark. “A20.1” and “PBS” represent negative control assays in which V_HHs were replaced with *C. difficile* toxin A-specific A20.1 V_HH[9] and PBS, respectively. V_HHs are color-coded according to their epitope bin designation (*see* Fig. 1d). Error bars indicate standard deviation (SD) of two technical replicates (**a**).

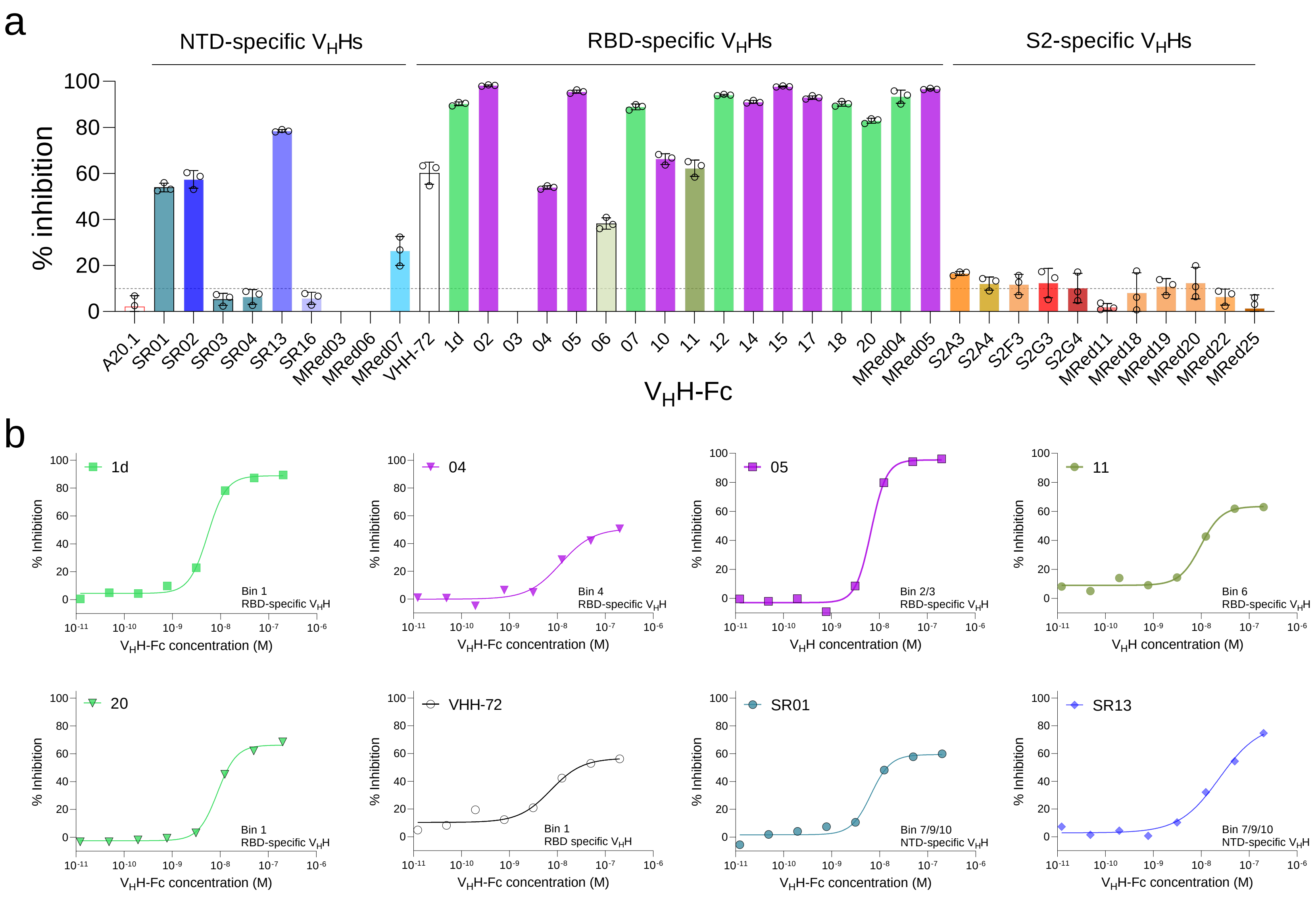
a
NTD-specific VHHs
RBD-specific VHHs
S2-specific VHHs
% inhibition
VHH-Fc
A20.1 SR01 SR02 SR03 SR04 SR13 SR16 MRed03 MRed06 MRed07 VHH-72 1d 02 03 04 05 06 07 10 11 12 14 15 17 18 20 MRed04 MRed05 S2A3 S2A4 S2F3 S2G3 S2G4 MRed11 MRed18 MRed19 MRed20 MRed22 MRed25
b
1d
Bin 1
RBD-specific VHH
04
Bin 4
RBD-specific VHH
05
Bin 2/3
RBD-specific VHH
11
Bin 6
RBD-specific VHH
20
Bin 1
RBD-specific VHH
VHH-72
Bin 1
RBD specific VHH
SR01
Bin 7/9/10
NTD-specific VHH
SR13
Bin 7/9/10
NTD-specific VHH
% Inhibition
VHH-Fc concentration (M)
VHH concentration (M)

Supplementary Figure 14. Flow cytometry SVNAs assessing the ability of bivalent SARS-CoV-2 V_HH-Fcs in blocking the binding of SARS-CoV-2 S to ACE2-expressing Vero E6 cells at 250 nM (a) or varying (b) V_HH-Fc concentrations. Representative plots are shown in **b**. 1d, 02, 04, 05, 11 and 20 V_HH-Fcs are RBD-specific, SR01 and SR13 are NTD-specific. The complete list of IC_{50}s calculated from graphs exemplified in **b** are reported in Table 2 and Fig. 4a. VHH-72 V_HH-Fc[7] is included as benchmark. “A20.1” is a negative control in which V_HH-Fcs were replaced with *C. difficile* toxin A-specific A20.1 V_HH-Fc[9]. Biotinylated SARS-CoV-2 Wuhan S was used in assays. V_HH-Fcs are color-coded according to their epitope bin designation (*see* Fig. 1d). Error bars indicate standard deviation (SD) of three technical replicates (**a**).

a

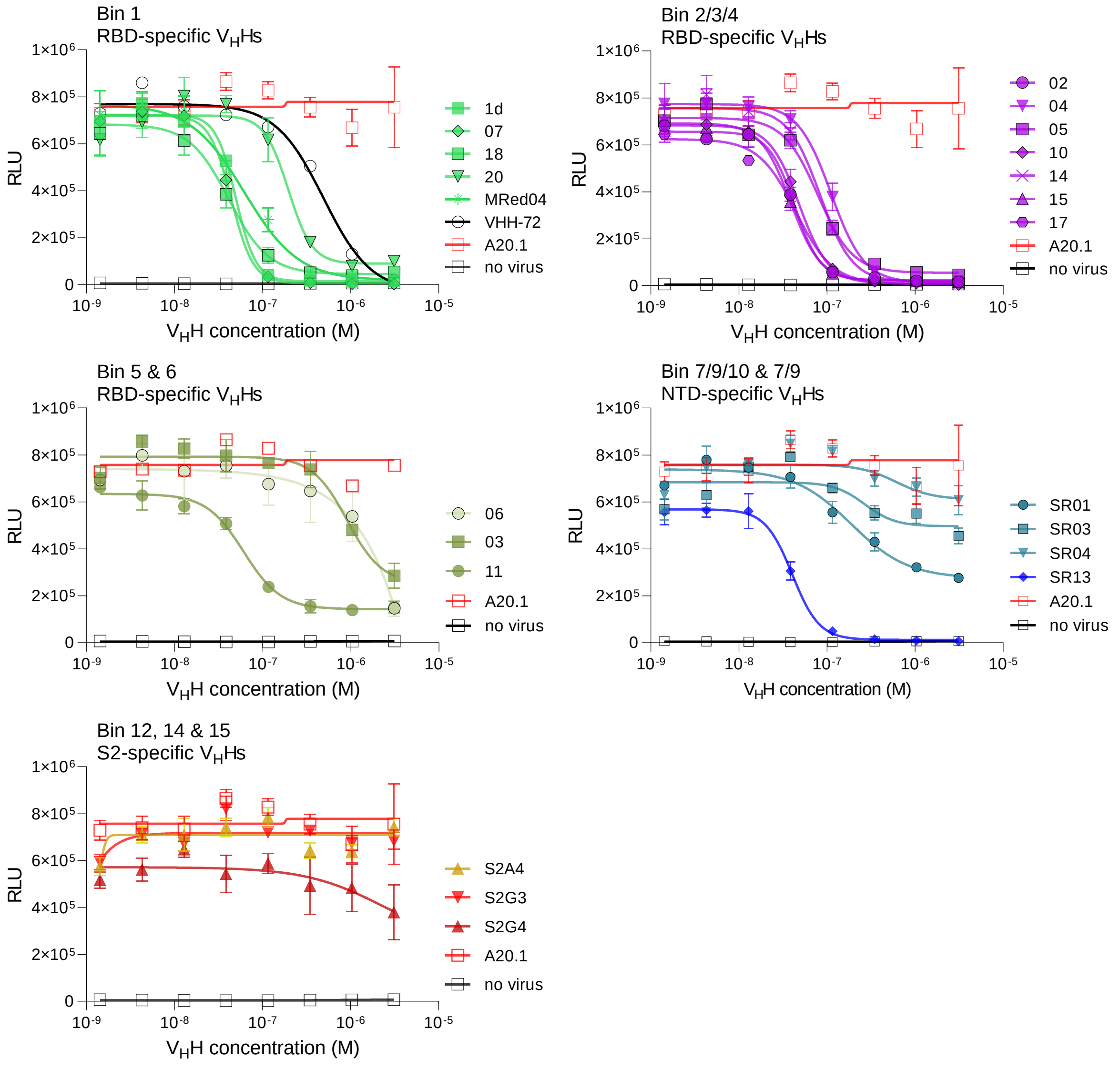
Bin 1
RBD-specific V_HHs
1×10^6
8×10^5
6×10^5
4×10^5
2×10^5
0
RLU
10^-9
10^-8
10^-7
10^-6
10^-5
V_HH concentration (M)
1d
07
18
20
MRed04
VHH-72
A20.1
no virus
Bin 2/3/4
RBD-specific V_HHs
02
04
05
10
14
15
17
A20.1
no virus
Bin 5 & 6
RBD-specific V_HHs
06
03
11
A20.1
no virus
Bin 7/9/10 & 7/9
NTD-specific V_HHs
SR01
SR03
SR04
SR13
A20.1
no virus
Bin 12, 14 & 15
S2-specific V_HHs
S2A4
S2G3
S2G4
A20.1
no virus

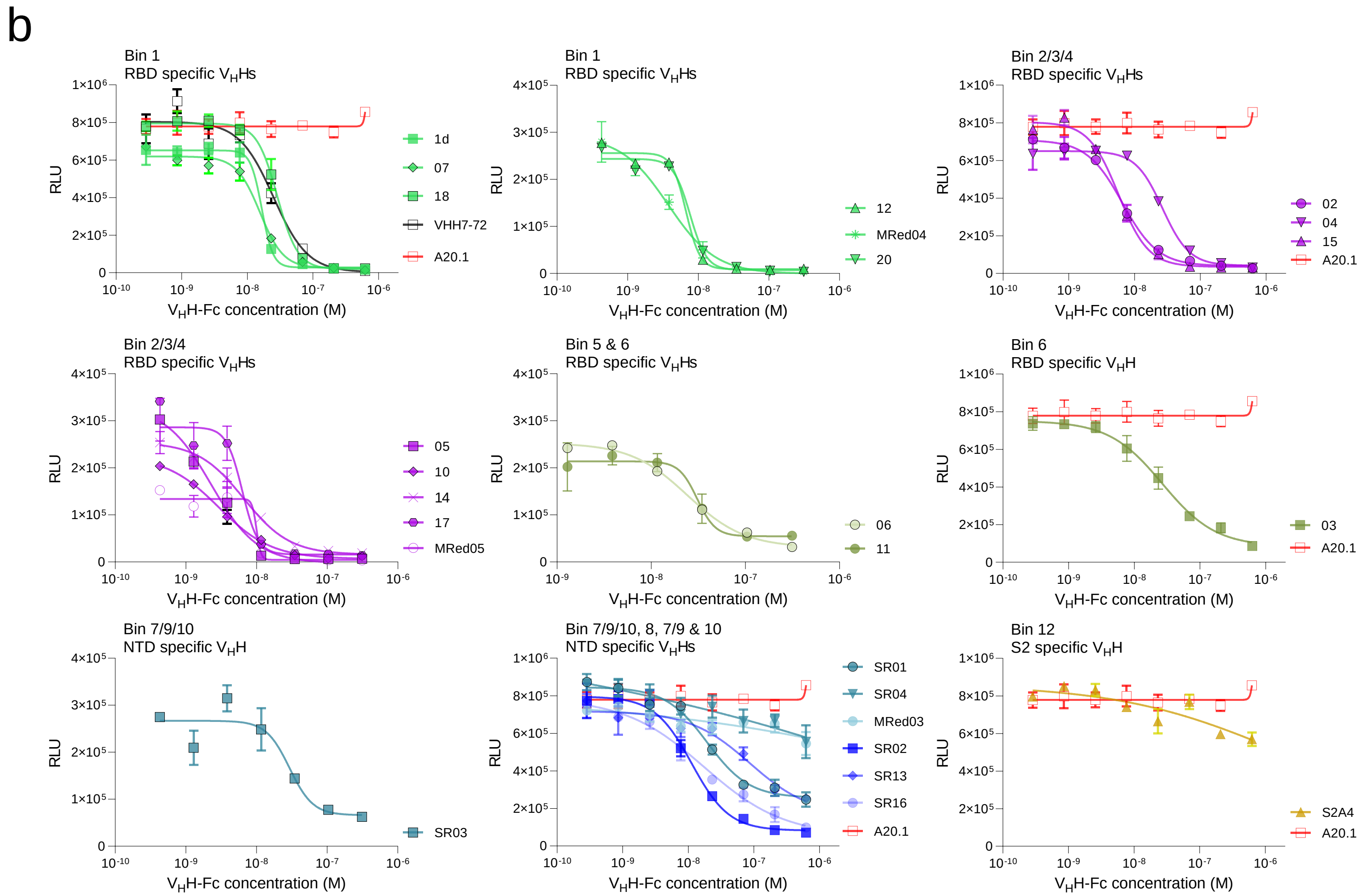
b
Bin 1
RBD specific V_HHs
1d
07
18
VHH7-72
A20.1
Bin 1
RBD specific V_HHs
12
MRed04
20
Bin 2/3/4
RBD specific V_HHs
02
04
15
A20.1
Bin 2/3/4
RBD specific V_HHs
05
10
14
17
MRed05
Bin 5 & 6
RBD specific V_HHs
06
11
Bin 6
RBD specific V_HH
03
A20.1
Bin 7/9/10
NTD specific V_HH
SR03
Bin 7/9/10, 8, 7/9 & 10
NTD specific V_HHs
SR01
SR04
MRed03
SR02
SR13
SR16
A20.1
Bin 12
S2 specific V_HH
S2A4
A20.1
RLU
V_HH-Fc concentration (M)

Supplementary Figure 15. Pseudotyped virus neutralization assays (PVNAs) assessing the ability of SARS-CoV-2 V_HHs (a) and V_HH-Fcs (b) in blocking the infection of ACE2-expressing HEK293T cells by SARS-CoV-2 spike-pseudotyped lentiviruses. Results, obtained from assays performed at multiple antibody concentrations, are shown only for those V_HHs/V_HH-Fcs that were positive at a threshold inhibition of 75% in preliminary inhibition screening assays. The complete list of IC_{50}s calculated from graphs are recorded in Table 2 and Fig. 4a. VHH-72 V_HH-Fc[7] is included as benchmark. "A20.1" represent negative antibody control assays in which SARS-CoV-2 V_HHs/V_HH-Fcs were replaced with *C. difficile* toxin A-specific A20.1 V_HH/V_HH-Fc[9], and provide upper limit infection RLU (relative light unit). "no virus" represent control assays in which pseudo-typed virus is omitted, and provide lower limit infection RLU. V_HHs/V_HH-Fcs are color-coded according to their epitope bin designation (*see* Fig. 1d). Error bars indicate standard error of the mean (SEM) of three technical replicates.

a

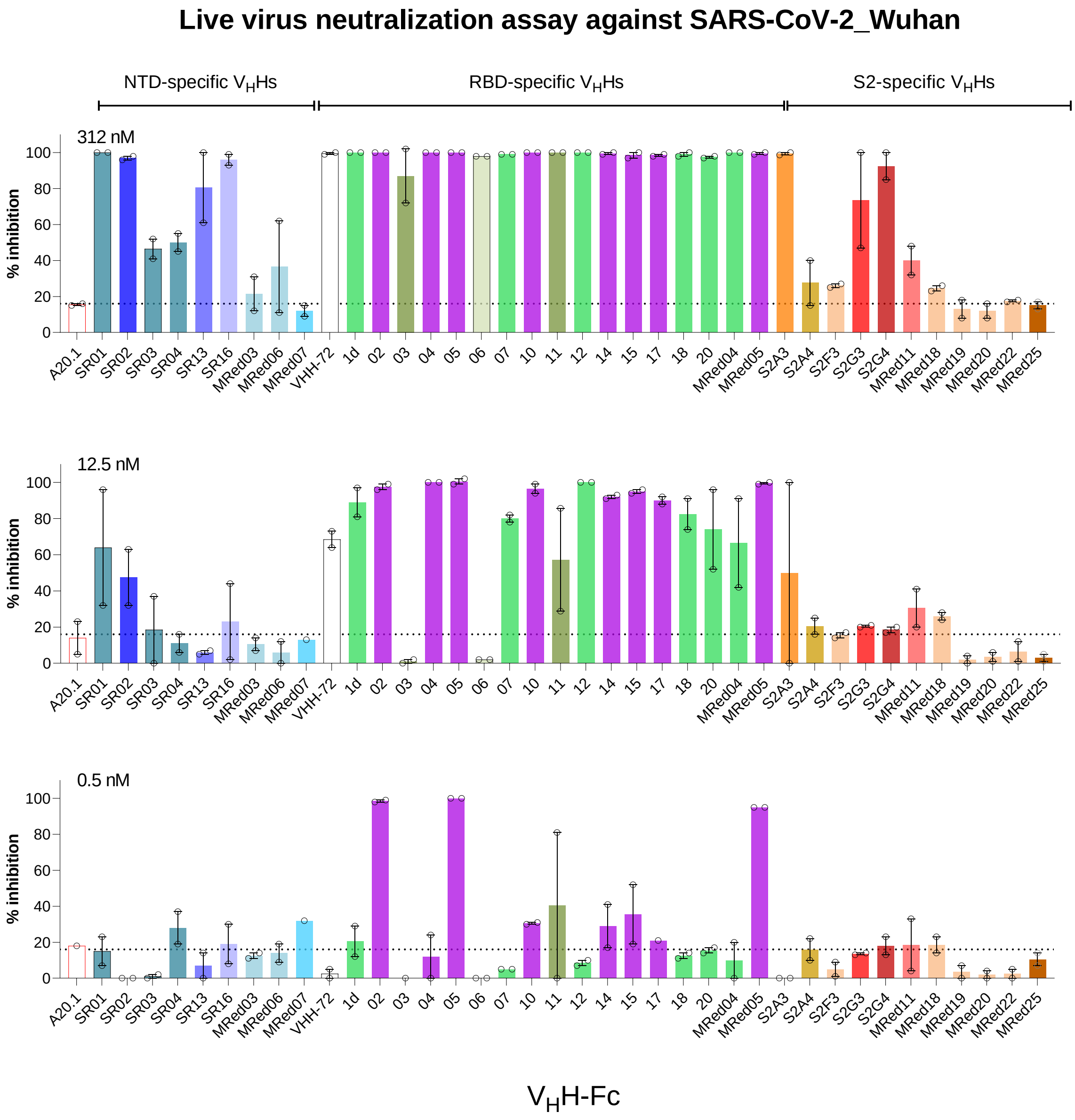

Live virus neutralization assay against SARS-CoV-2_Wuhan
NTD-specific V_HHs
RBD-specific V_HHs
S2-specific V_HHs
312 nM
12.5 nM
0.5 nM
% inhibition
0
20
40
60
80
100
A20.1
SR01
SR02
SR03
SR04
SR13
SR16
MRed03
MRed06
MRed07
VHH-72
1d
02
03
04
05
06
07
10
11
12
14
15
17
18
20
MRed04
MRed05
S2A3
S2A4
S2F3
S2G3
S2G4
MRed11
MRed18
MRed19
MRed20
MRed22
MRed25
V_HH-Fc

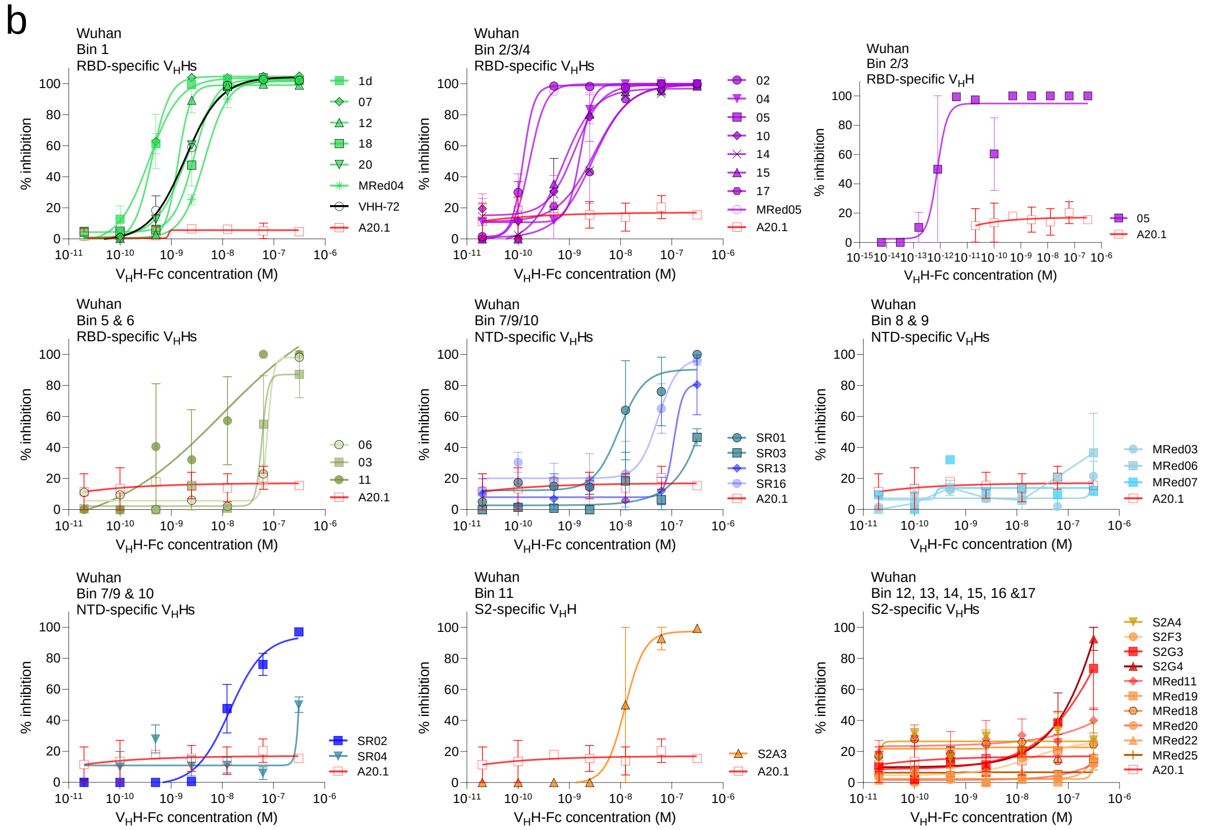

Supplementary Figure 16. Live virus neutralization assays (LVNAs) assessing the ability of SARS-CoV-2 VHH-Fcs in blocking the infection of ACE2-expressing Vero E6 cells by SARS-CoV-2 Wuhan variant at fixed (a) or varying (b) VHH-Fc concentrations. a Inhibition assays were performed at 312, 12.5 or 0.5 nM VHH-Fc concentrations. *IC*50s calculated from graphs in **b** are reported in Table 2, Fig 4a and Fig. 4c. VHH-72[7] and *C. difficile* toxin A-specific VHH A20.1[9] are included as a benchmark and negative antibody control, respectively. VHH-Fcs are color-coded according to their epitope bin designation (*see* Fig. 1d). Error bars indicate standard error of mean (SEM) of two technical replicates.

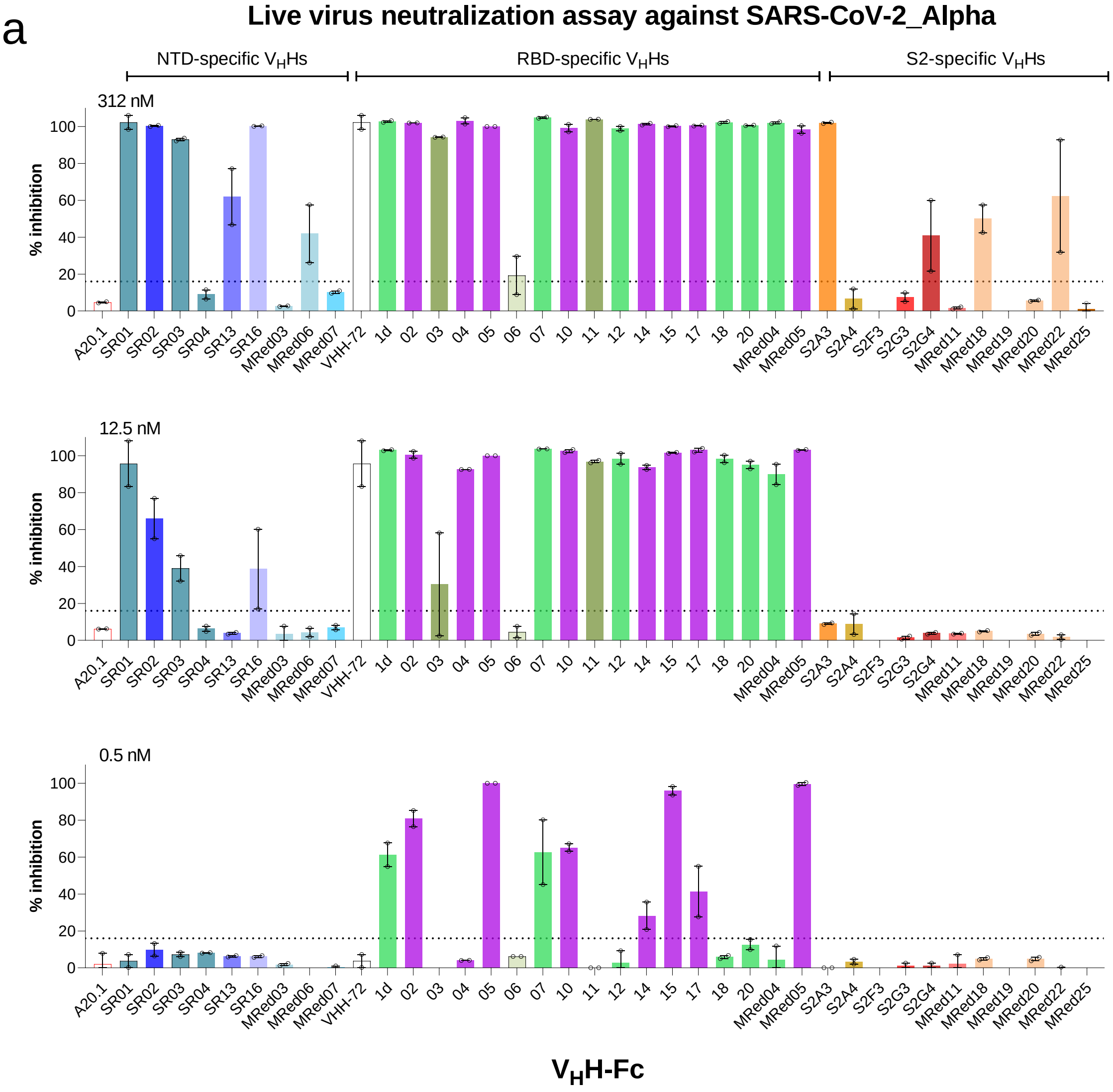
a
Live virus neutralization assay against SARS-CoV-2_Alpha
NTD-specific V_HHs
RBD-specific V_HHs
S2-specific V_HHs
312 nM
12.5 nM
0.5 nM
% inhibition
A20.1 SR01 SR02 SR03 SR04 SR13 SR16 MRed03 MRed06 MRed07 VHH-72 1d 02 03 04 05 06 07 10 11 12 14 15 17 18 20 MRed04 MRed05 S2A3 S2A4 S2F3 S2G3 S2G4 MRed11 MRed18 MRed19 MRed20 MRed22 MRed25
V_HH-Fc

b

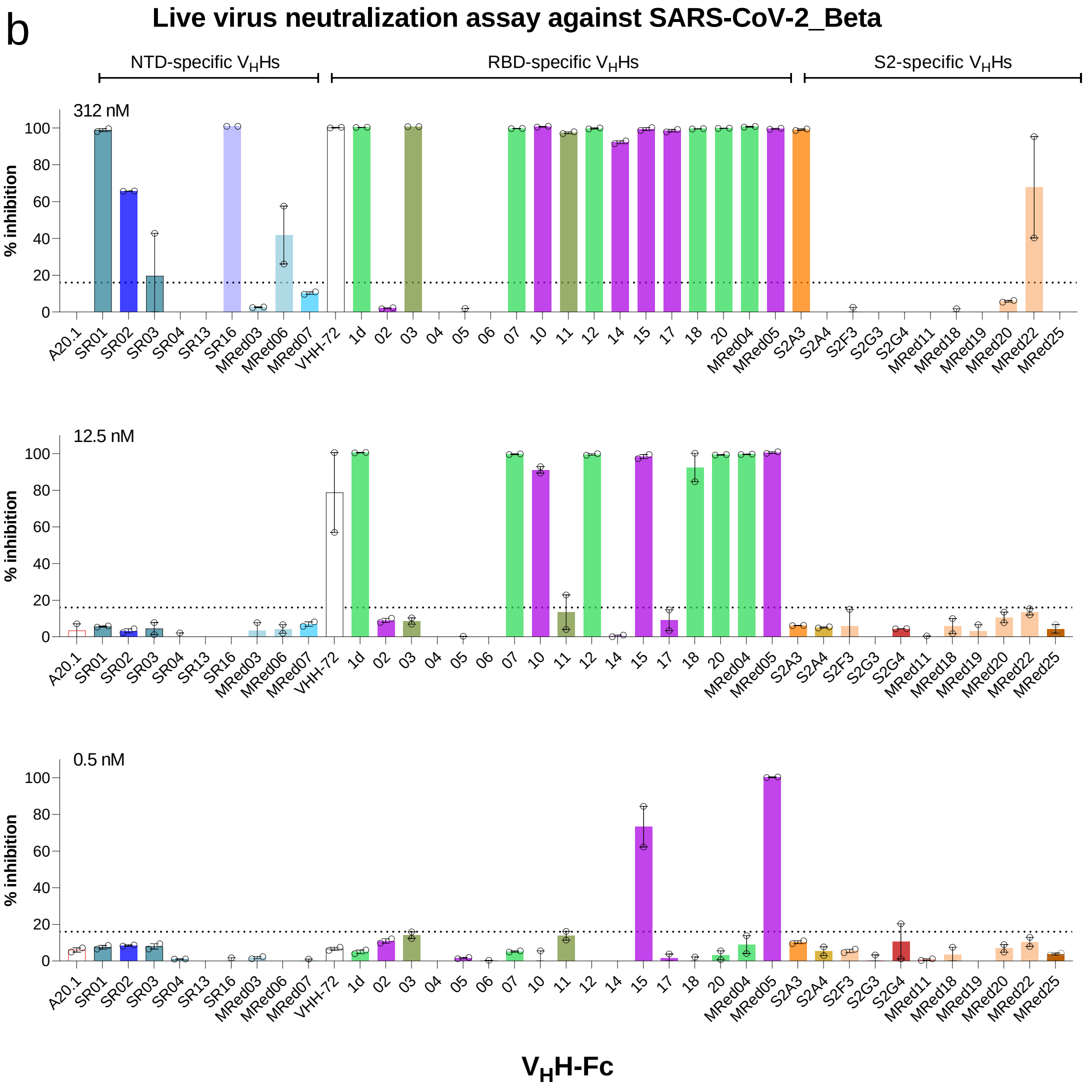

Live virus neutralization assay against SARS-CoV-2_Beta
NTD-specific V_HHs
RBD-specific V_HHs
S2-specific V_HHs
312 nM
12.5 nM
0.5 nM
% inhibition
0
20
40
60
80
100
A20.1
SR01
SR02
SR03
SR04
SR13
SR16
MRed03
MRed06
MRed07
VHH-72
1d
02
03
04
05
06
07
10
11
12
14
15
17
18
20
MRed04
MRed05
S2A3
S2A4
S2F3
S2G3
S2G4
MRed11
MRed18
MRed19
MRed20
MRed22
MRed25
V_HH-Fc

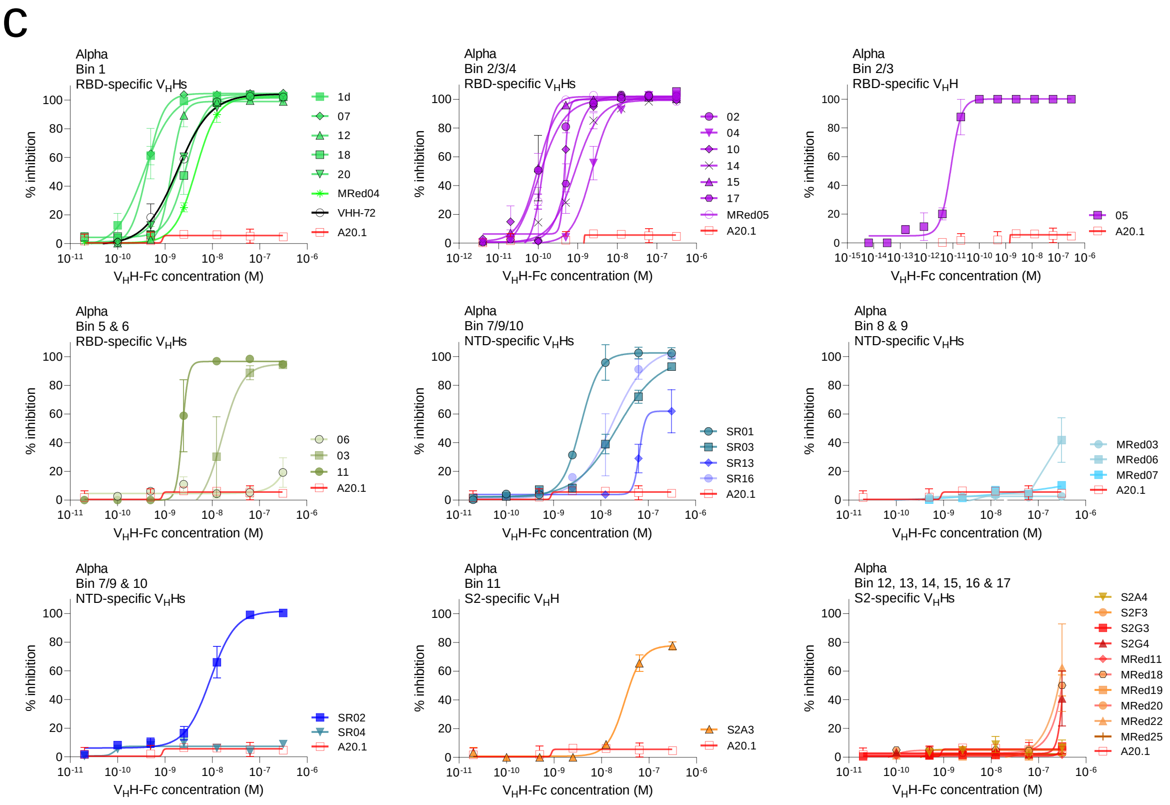
C
Alpha
Bin 1
RBD-specific VHHs
1d
07
12
18
20
MRed04
VHH-72
A20.1
Alpha
Bin 2/3/4
RBD-specific VHHs
02
04
10
14
15
17
MRed05
A20.1
Alpha
Bin 2/3
RBD-specific VHH
05
A20.1
Alpha
Bin 5 & 6
RBD-specific VHHs
06
03
11
A20.1
Alpha
Bin 7/9/10
NTD-specific VHHs
SR01
SR03
SR13
SR16
A20.1
Alpha
Bin 8 & 9
NTD-specific VHHs
MRed03
MRed06
MRed07
A20.1
Alpha
Bin 7/9 & 10
NTD-specific VHHs
SR02
SR04
A20.1
Alpha
Bin 11
S2-specific VHH
S2A3
A20.1
Alpha
Bin 12, 13, 14, 15, 16 & 17
S2-specific VHHs
S2A4
S2F3
S2G3
S2G4
MRed11
MRed18
MRed19
MRed20
MRed22
MRed25
A20.1
% inhibition
VHH-Fc concentration (M)

d

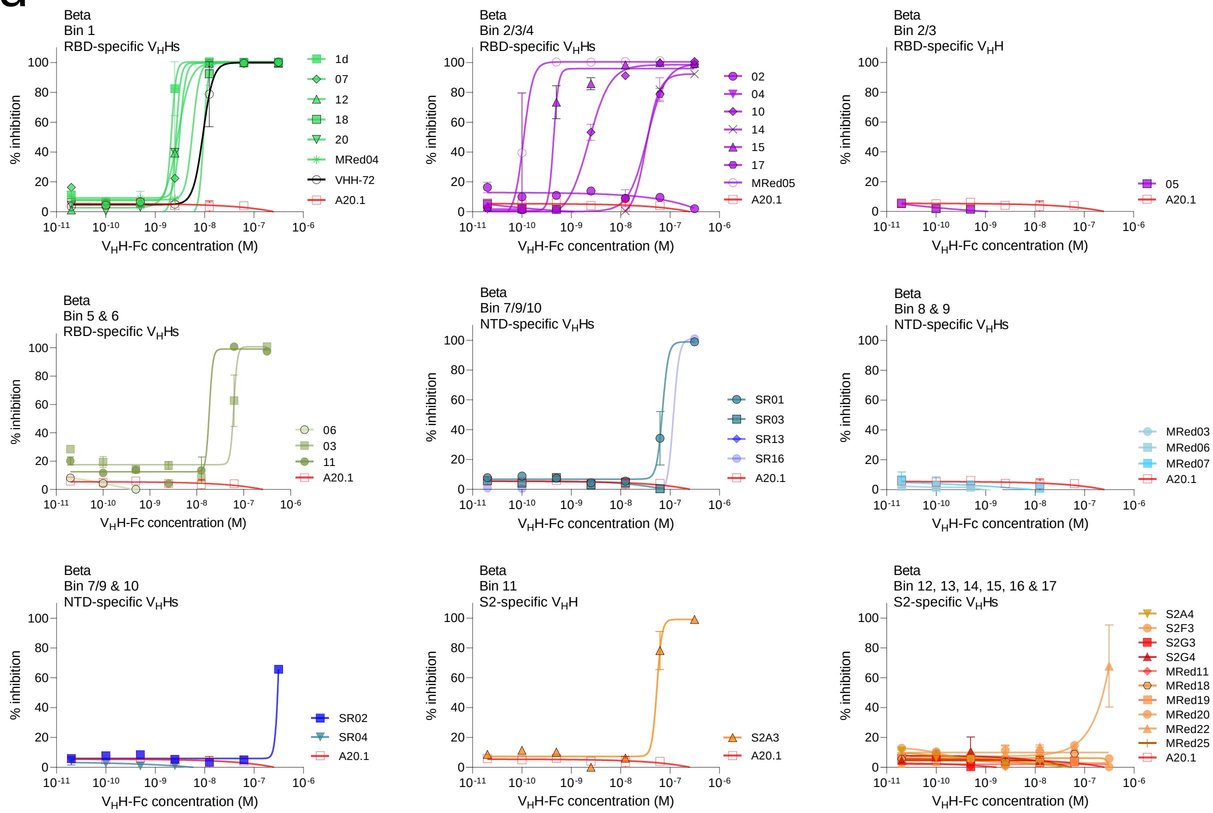

Supplementary Figure 17. LVNAs assessing the ability of SARS-CoV-2 V_HH-Fcs in blocking the infection of ACE2-expressing Vero E6 cells by SARS-CoV-2 Alpha (a and c) and Beta (b and d) variants at fixed (a and b) or varying (c and d) V_HH-Fc concentrations. a & **b** Inhibition assays were performed at 312, 12.5 or 0.5 nM V_HH-Fc concentrations. IC_{50}s calculated from graphs in **c** and **d** are recorded in Table 2 and Fig. 4c. VHH-72[7] and *C. difficile* toxin A-specific V_HH A20.1[9] are included as a benchmark and negative antibody control, respectively. V_HH-Fcs are color-coded according to their epitope bin designation (*see* Fig. 1d). Error bars indicate standard error of mean (SEM) of two technical replicates.

a

Live virus neutralization assay against SARS-CoV-2_Omicron

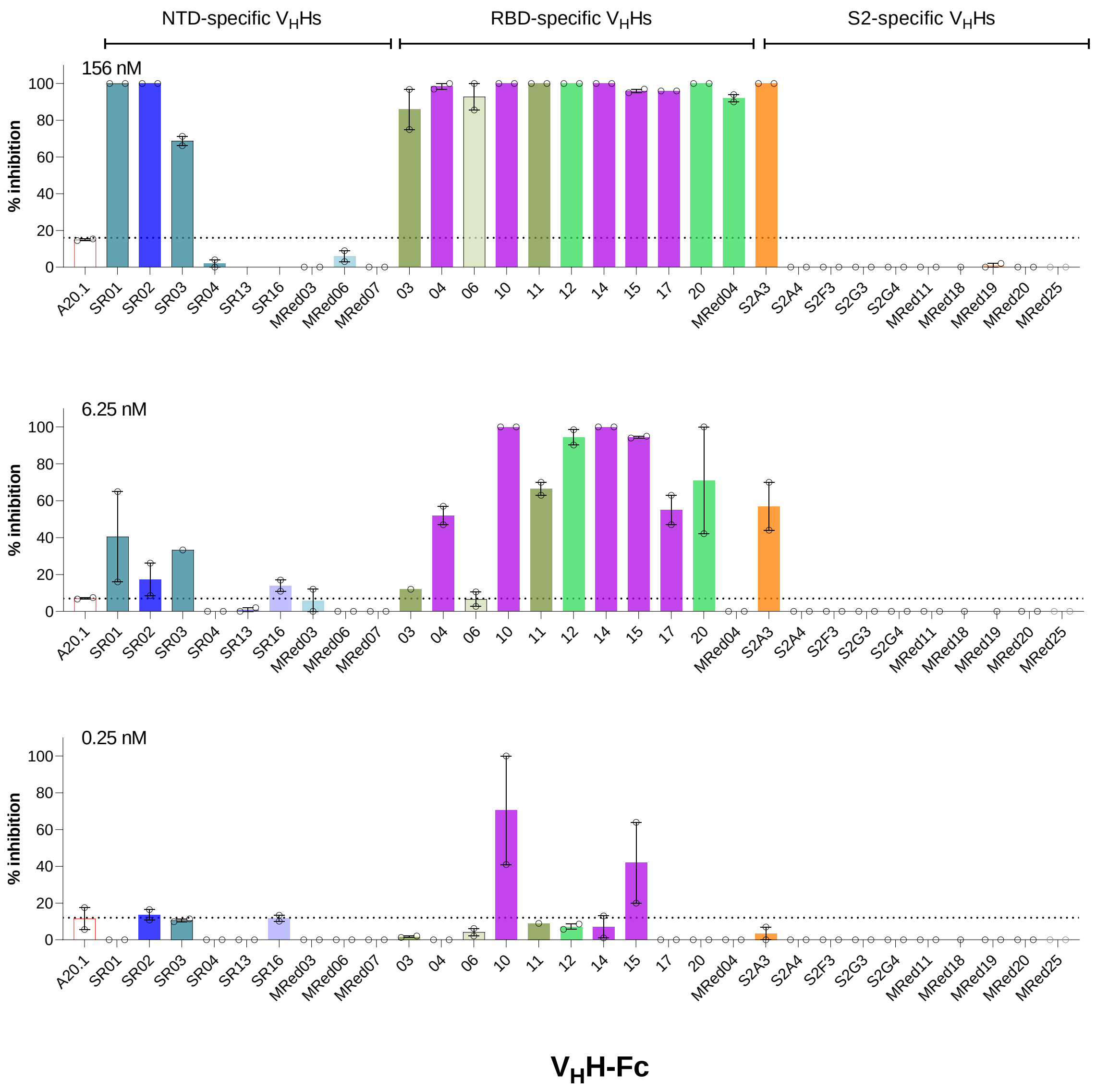

b

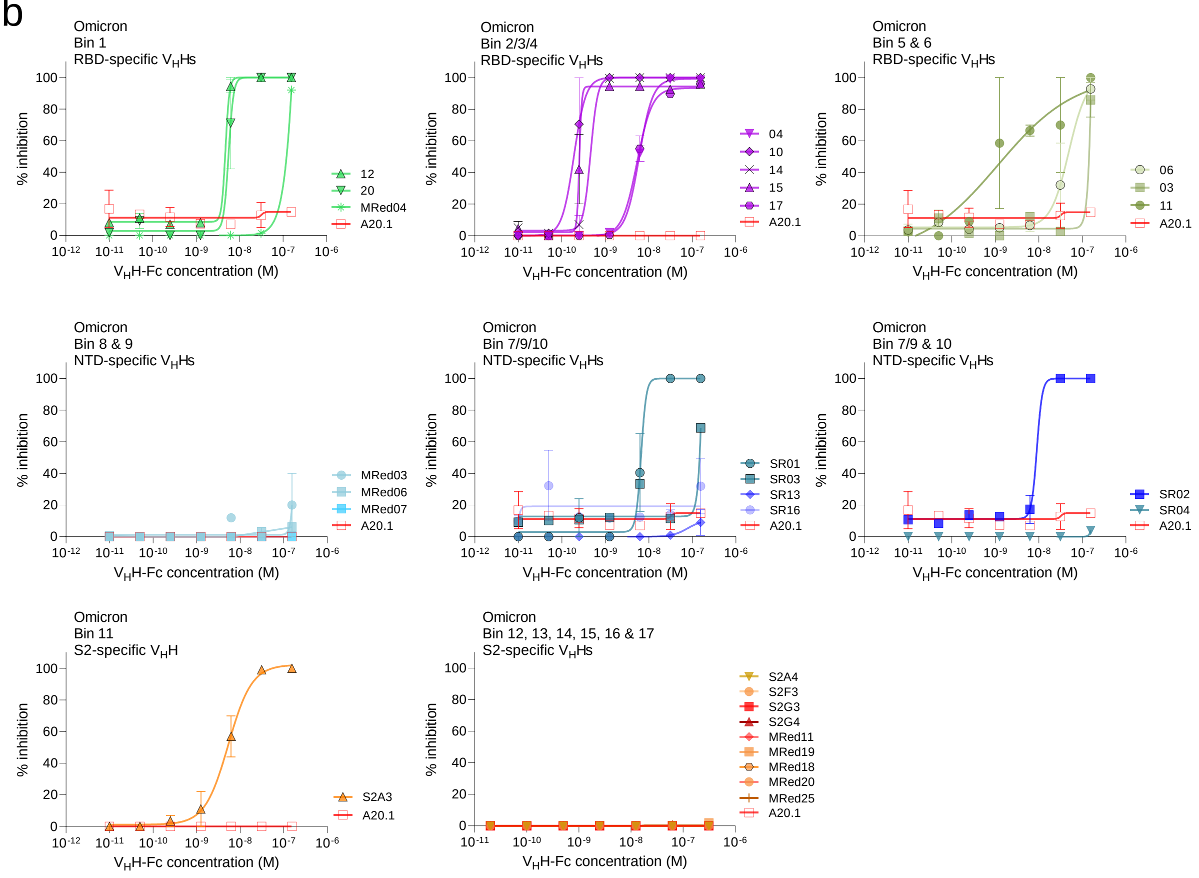

Supplementary Figure 18. LVNAs assessing the ability of SARS-CoV-2 V_HH-Fcs in blocking the infection of ACE2-expressing Vero E6 cells by SARS-CoV-2 Omicron B.1.1.529 variant at fixed (a) or varying (b) V_HH-Fc concentrations. a Inhibition assays were performed at 156, 6.25 or 0.25 nM V_HH-Fc concentrations using V_HH-Fcs that were positive for binding to Omicron S (Fig. 2a). IC_{50}s calculated from graphs in **b** are recorded in Table 2 and Fig. 4c. *C. difficile* toxin A-specific V_HH A20.1[9] is included as negative antibody control. V_HH-Fcs are color-coded according to their epitope bin designation (*see* Fig. 1d). Error bars indicate standard error of mean (SEM) of two technical replicates.

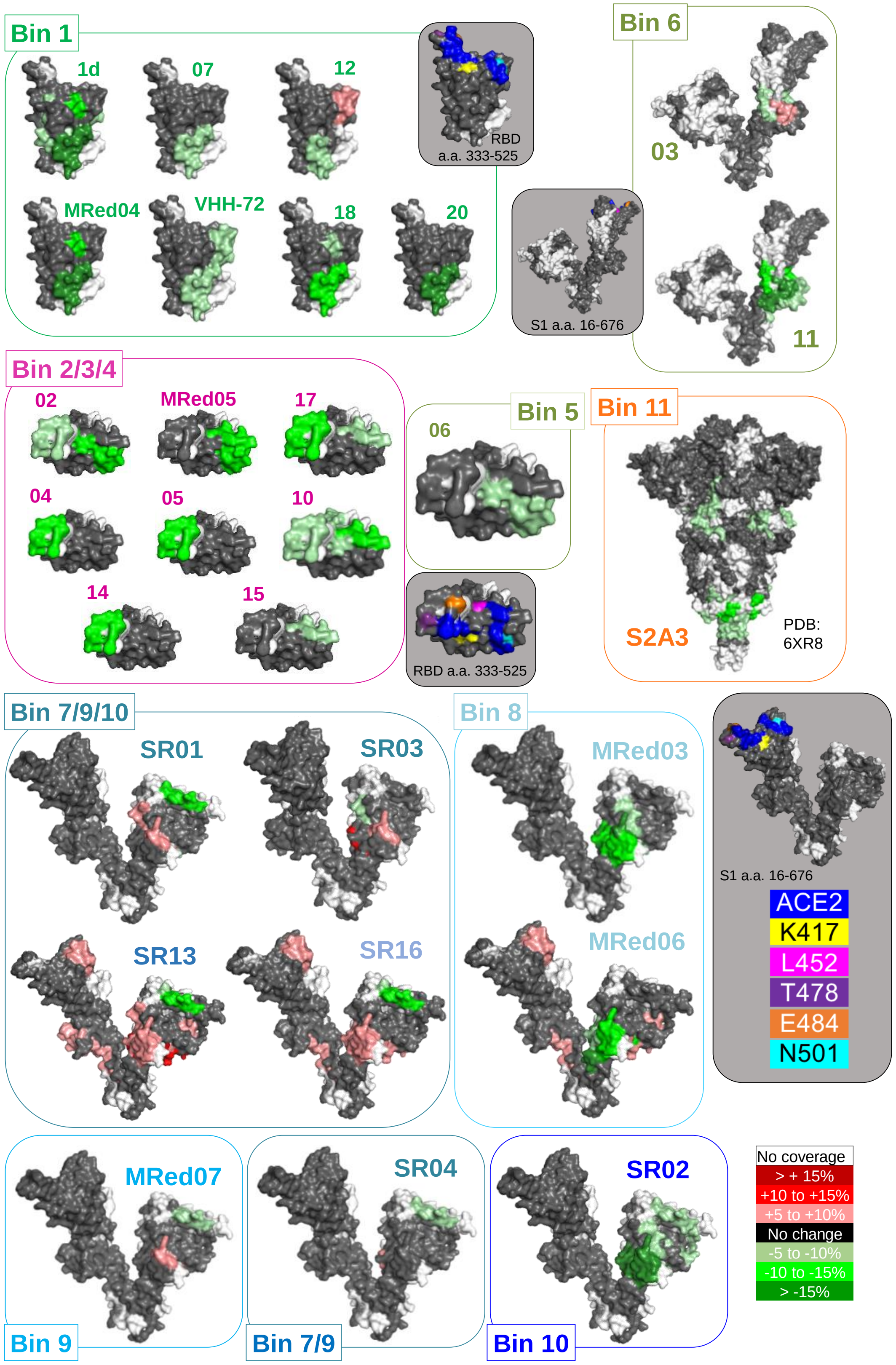
Bin 1
1d
07
12
MRed04
VHH-72
18
20
RBD
a.a. 333-525
S1 a.a. 16-676
Bin 6
03
11
Bin 2/3/4
02
MRed05
17
04
05
10
14
15
Bin 5
06
RBD a.a. 333-525
Bin 11
S2A3
PDB:
6XR8
Bin 7/9/10
SR01
SR03
SR13
SR16
Bin 8
MRed03
MRed06
S1 a.a. 16-676
ACE2
K417
L452
T478
E484
N501
MRed07
Bin 9
SR04
Bin 7/9
SR02
Bin 10
No coverage
> + 15%
+10 to +15%
+5 to +10%
No change
-5 to -10%
-10 to -15%
> -15%

Supplementary Figure 19. Visualization of differential HDX. ΔD measurements (D_{bound}-$D_{control}$) projected onto three-dimensional structures (PDB 6XR8). Bins are colored based on Fig. 1d. Stabilizations are shown in green, and destabilizations in red, while regions with no significant changes in deuteration are shown in grey and missing coverage in white. Key structural features are highlighted in shaded grey boxes. Here, the ACE2 binding site is shown in blue, and five mutations from VoCs over the RBD and S1 domain are included for reference (*see* legend).

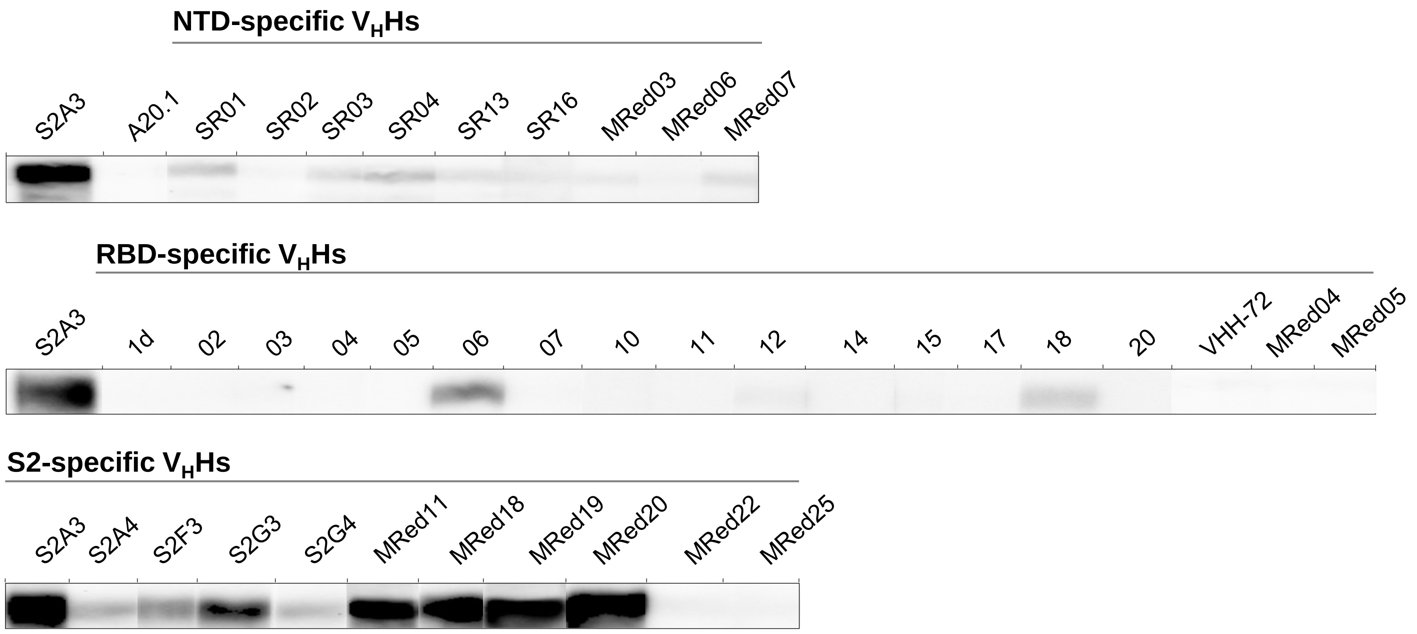

Supplementary Figure 20. Epitope typing of SARS-CoV-2 V_HHs by SDS-PAGE/western blot against denatured S. Presence of blots (binding signals) indicate V_HH-Fcs recognizes a linear epitope. The absence of binding signals is an indirect indication of V_HH-Fcs recognizing conformational epitopes. A20.1[9] V_HH-Fc was included as isotype control. As expected, the conformational epitope-specific VHH-72[7] shows no binding towards denatured S. S2A3 was included in each row as reference. Epitope types are reported in Table 1.

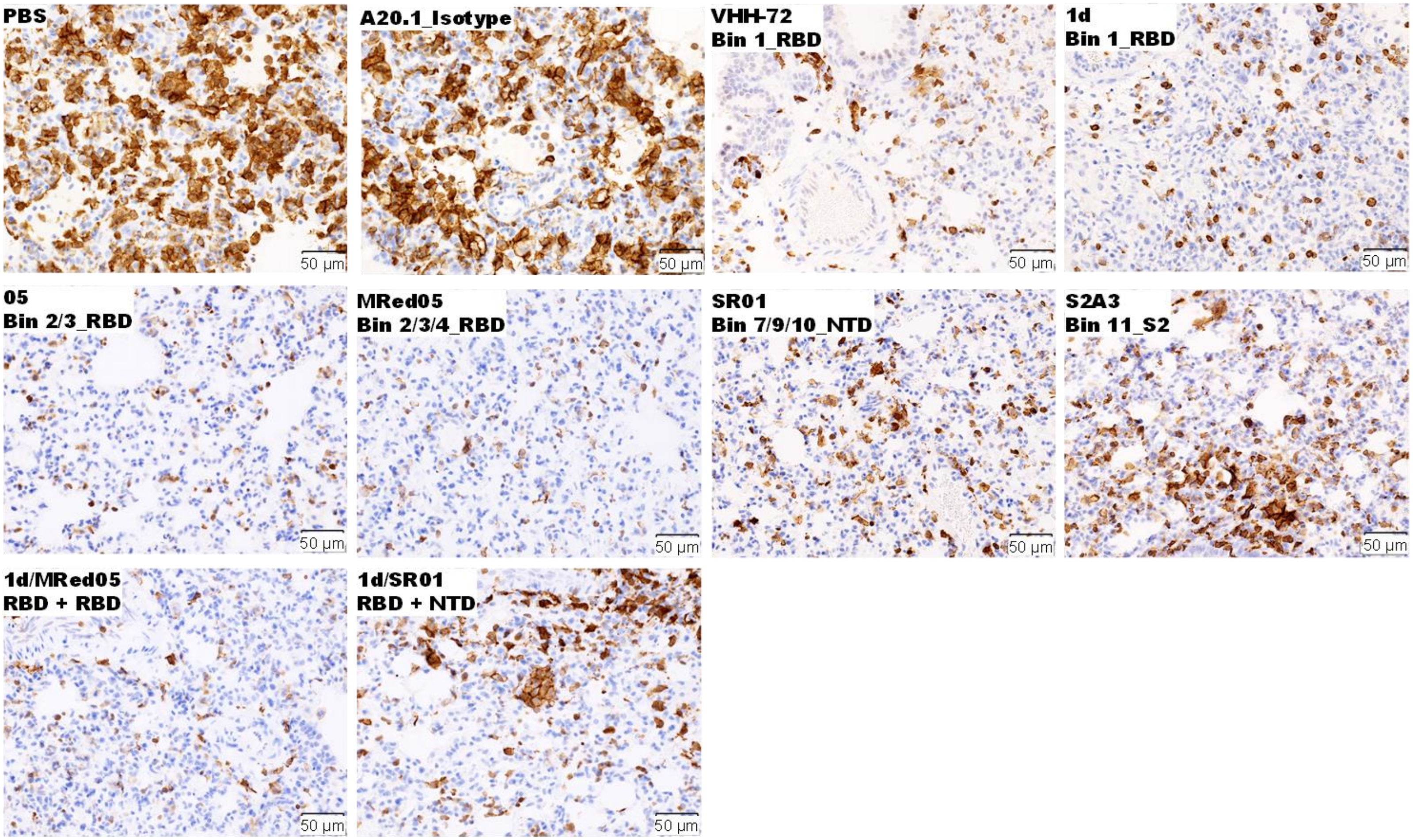

Supplementary Figure 21. Immunohistochemical detection of infiltrating macrophages in the lungs of V_HH-Fc-treated animals. Untreated (PBS) and A20.1[9] isotype-treated animals showed an intense immune reaction to anti-Iba-1 antibody and an increased number of Iba-1-positive macrophages in the consolidated areas. A substantial reduction in the number of Iba-1-positive macrophages was seen in the perivascular areas and pulmonary interstitium in the lungs of V_HH-Fc-treated animals. Representative images are shown from a single experiment.

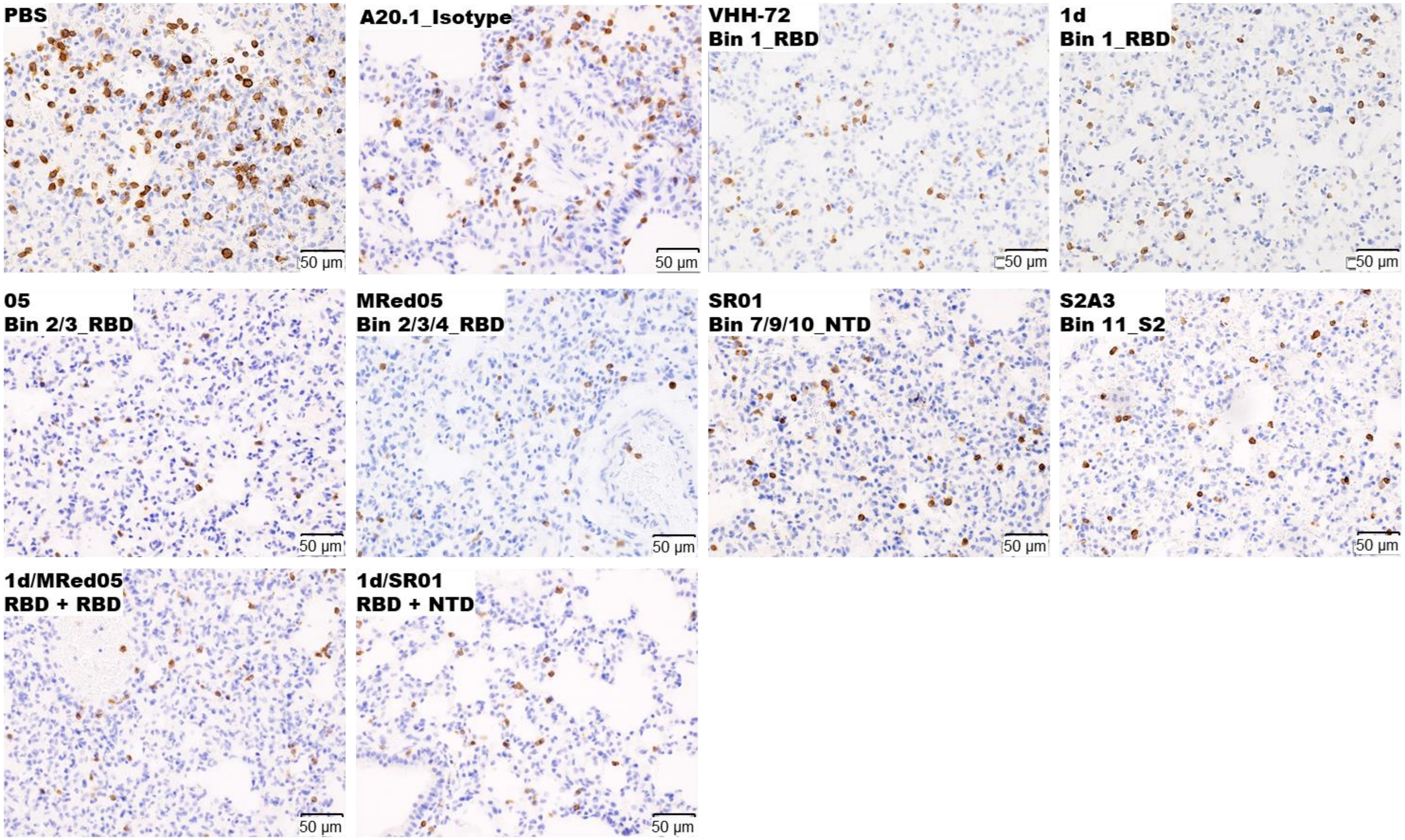

Supplementary Figure 22. Immunohistochemical detection of T lymphocytes in the lungs of V_HH-Fc-treated animals. Untreated (PBS) and A20.1[9] isotype-treated animals showed an increased number of T lymphocytes in the pulmonary interstitium. A dramatic decrease in the number of T lymphocytes was seen in the lungs of V_HH-Fc-treated animals. Representative images are shown from a single experiment.

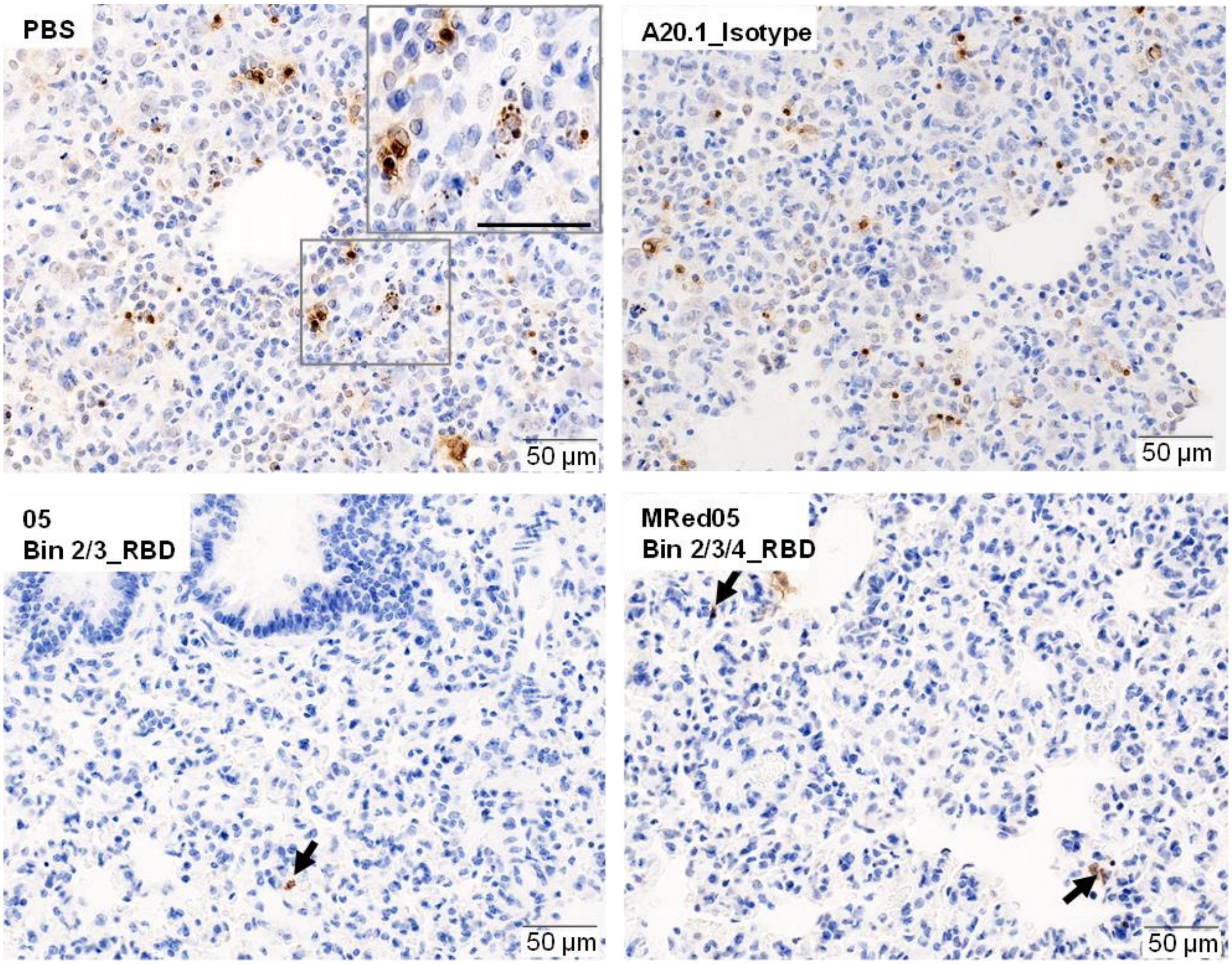

Supplementary Figure 23. Immunohistochemical detection of apoptotic cells in the lungs of V_HH-Fc treated animals. Untreated (PBS) and A20.1[9] isotype-treated animals showed an increase in the number of TUNEL-positive cells with classical features of apoptotic cells in the pulmonary interstitium. The large grey frame in the corner of PBS panel shows the magnification of the region (small grey frame) in the lung parenchyma, scale bar = 50 µm. A marked reduction in the TUNEL-positive cells was seen in the lungs of 05- and MRed05-treated animals. Black arrows indicate occasional TUNEL-positive cells. Representative images are shown from a single experiment.

Supplementary references

1. Akache B*, et al.* Immunogenic and efficacious SARS-CoV-2 vaccine based on resistin-trimerized spike antigen SmT1 and SLA archaeosome adjuvant. *Sci Rep* **11**, 21849 (2021).

2. Wrapp D*, et al.* Cryo-EM structure of the 2019-nCoV spike in the prefusion conformation. *Science* **367**, 1260-1263 (2020).

3. Colwill K*, et al.* A scalable serology solution for profiling humoral immune responses to SARS-CoV-2 infection and vaccination. *Clin & transl immunol* **11**, e1380 (2022).

4. Galipeau Y*, et al.* Relative ratios of human seasonal coronavirus antibodies predict the efficiency of cross-enutralization of SARS-CoV-2 spike binding to ACE2. *EBioMedicine* **74**, 103700 (2021).

5. Stuible M*, et al.* Rapid, high-yield production of full-length SARS-CoV-2 spike ectodomain by transient gene expression in CHO cells. *J Biotechnol* **326**, 21-27 (2021).

6. Sulea T*, et al.* Structure-based dual affinity optimization of a SARS-CoV-1/2 cross-reactive single-domain antibody. *PloS one* **17**, e0266250 (2022).

7. Wrapp D*, et al.* Structural basis for potent neutralization of betacoronaviruses by single-domain camelid antibodies. *Cell* **181**, 1004-1015 e1015 (2020).

8. Rossotti MA*, et al.* Camelid single-domain antibodies raised by DNA immunization are potent inhibitors of EGFR signaling. *Biochem J* **476**, 39-50 (2019).

9. Hussack G, *et al*. Neutralization of *Clostridium difficile* toxin A with single-domain antibodies targeting the cell receptor binding domain. *J Biol Chem* **286**, 8961-8976 (2011).
