# Supplementary material for: Arsenal of nanobodies shows broad-spectrum neutralization against SARS-CoV-2 variants of concern in vitro and in vivo in hamster models
Source: Commun Biol. 2022 Sep 9;5:933. doi: 10.1038/s42003-022-03866-z (PMC9461429; doi:10.1038/s42003-022-03866-z)
Supplement: Supplementary file 2 — Description of Additional Supplementary Files [file 42003_2022_3866_MOESM2_ESM.pdf]

Description of Additional Supplementary Files

File name: Supplementary Data 1
Description: The source data behind the main figures in the paper.
