# Supplementary material for: Arsenal of nanobodies shows broad-spectrum neutralization against SARS-CoV-2 variants of concern in vitro and in vivo in hamster models
Source: Commun Biol. 2022 Sep 9;5:933. doi: 10.1038/s42003-022-03866-z (PMC9461429; doi:10.1038/s42003-022-03866-z)
Supplement: Supplementary file 4 — Reporting Summary [file 42003_2022_3866_MOESM4_ESM.pdf]

nature portfolio

Corresponding author(s): DBPR/COMMSBIO-22-0038-B

Last updated by author(s): Aug 7, 2022

Reporting Summary

Nature Portfolio wishes to improve the reproducibility of the work that we publish. This form provides structure for consistency and transparency in reporting. For further information on Nature Portfolio policies, see our Editorial Policies and the Editorial Policy Checklist.

Statistics

For all statistical analyses, confirm that the following items are present in the figure legend, table legend, main text, or Methods section.

n/a	Confirmed	
☐	☒	The exact sample size (*n*) for each experimental group/condition, given as a discrete number and unit of measurement
☐	☒	A statement on whether measurements were taken from distinct samples or whether the same sample was measured repeatedly
☐	☒	The statistical test(s) used AND whether they are one- or two-sided<br>*Only common tests should be described solely by name; describe more complex techniques in the Methods section.*
☒	☐	A description of all covariates tested
☒	☐	A description of any assumptions or corrections, such as tests of normality and adjustment for multiple comparisons
☐	☒	A full description of the statistical parameters including central tendency (e.g. means) or other basic estimates (e.g. regression coefficient) AND variation (e.g. standard deviation) or associated estimates of uncertainty (e.g. confidence intervals)
☐	☒	For null hypothesis testing, the test statistic (e.g. *F*, *t*, *r*) with confidence intervals, effect sizes, degrees of freedom and *P* value noted<br>*Give P values as exact values whenever suitable.*
☒	☐	For Bayesian analysis, information on the choice of priors and Markov chain Monte Carlo settings
☒	☐	For hierarchical and complex designs, identification of the appropriate level for tests and full reporting of outcomes
☒	☐	Estimates of effect sizes (e.g. Cohen's *d*, Pearson's *r*), indicating how they were calculated

Our web collection on statistics for biologists contains articles on many of the points above.

Software and code

Policy information about availability of computer code

Data collection	Hydrogen-deuterium exchange mass spectrometry, Waters Synapt G2-Si Mass Spectrometer.
Data analysis	Flow cytometry, FlowJo; SPR sensorgrams, BIAevaluation Software v3.0 (Cytiva); Hydrogen-deuterium exchange mass spectrometry, HDExaminer v2 (Sierra Analytics), MS Studio; Spike phylogenetic tree data, MEGA11 (https://www.megasoftware.net/citations); ELISA data analysis, Microsoft Excel Standard 2013; various curve fittings and graphs for VHH CDR3 diversity, ELISA, cell binding, neutralization, stability and animal studies data, GraphPad Prism 8.2.1 and 9.1.2.

For manuscripts utilizing custom algorithms or software that are central to the research but not yet described in published literature, software must be made available to editors and reviewers. We strongly encourage code deposition in a community repository (e.g. GitHub). See the Nature Portfolio guidelines for submitting code & software for further information.

Data

Policy information about availability of data

All manuscripts must include a data availability statement. This statement should provide the following information, where applicable:

- Accession codes, unique identifiers, or web links for publicly available datasets
- A description of any restrictions on data availability
- For clinical datasets or third party data, please ensure that the statement adheres to our policy

The data generated and/or analyzed during the current study are available from the corresponding author upon request. The source data for figures and tables are provided in Supplementary Information and Supplementary Data 1.

Human research participants

Policy information about studies involving human research participants and Sex and Gender in Research.

Reporting on sex and gender	n/a
Population characteristics	n/a
Recruitment	n/a
Ethics oversight	n/a

Note that full information on the approval of the study protocol must also be provided in the manuscript.

Field-specific reporting

Please select the one below that is the best fit for your research. If you are not sure, read the appropriate sections before making your selection.

☒ Life sciences ☐ Behavioural & social sciences ☐ Ecological, evolutionary & environmental sciences

For a reference copy of the document with all sections, see nature.com/documents/nr-reporting-summary-flat.pdf

Life sciences study design

All studies must disclose on these points even when the disclosure is negative.

Sample size	For in vivo efficacy studies, power calculation was used to determine minimal sample size per group that will give a Power of 80% for a P of 0.05. The sample size was determined to be a minimum of 6 hamsters per group.
Data exclusions	No data were excluded from analyses.
Replication	The number of individual animals per group represents a biological replicate of lung viral titer; therefore, the plaque assay to determine lung viral load was not done as technical replicates.<br><br>For IHC experiments, for each animal, 4-5 images were taken and a representative image is shown. Results are from a single experiment.<br><br>The animal studies were not repeated.
Randomization	Animals in a single cohort were randomly assigned to each treatment group.
Blinding	For in vivo efficacy studies, each treatment group was assigned a numerical value, and animals within each group were randomly assigned another numerical value. These stand as IDs that were associated back to a master list. All subsequent manipulations and assays used these numerical ID. While the study was not completely blind, as each group ID was known, every effort was made to reduce unintentional bias during the conduct of each experiment.<br><br>IHC experiments were performed by experts who did not know the identity of the experimental groups.

Reporting for specific materials, systems and methods

We require information from authors about some types of materials, experimental systems and methods used in many studies. Here, indicate whether each material, system or method listed is relevant to your study. If you are not sure if a list item applies to your research, read the appropriate section before selecting a response.

Materials & experimental systems

n/a	Involved in the study	
☐	☒	Antibodies
☐	☒	Eukaryotic cell lines
☒	☐	Palaeontology and archaeology
☐	☒	Animals and other organisms
☒	☐	Clinical data
☒	☐	Dual use research of concern

Methods

n/a	Involved in the study	
☒	☐	ChIP-seq
☐	☒	Flow cytometry
☒	☐	MRI-based neuroimaging

Antibodies

Antibodies used

Anti-SARS-CoV-2 nanobodies, anti-EGFR NRCsdAb022 nanobody, anti-SARS-CoV-1 VHH-72 nanobody, anti-C. difficile toxin A A20.1 nanobody (all produced in house).

Goat Anti-Human IgG (Fc specific)–Peroxidase Antibody (Sigma, Cat#A0170)

Peroxidase AffiniPure Goat Anti-Rabbit IgG (H+L) (Jackson ImmunoResearch, Cat#111-035-144)
Goat anti-Llama IgG-Heavy and Light Chain Antibody HRP Conjugated (Bethyl Laboratories, Cat#A160-100P)
Anti-M13 Major Coat Protein Antibody (RL-ph1) HRP (Santa Cruz Biotechnology, Cat#SC-53004HRP)

AffiniPure Goat Anti-Human IgG, Fcγ fragment specific (Jackson ImmunoResearch, Cat#109-005-098)

R-Phycoerythrin AffiniPure F(ab')$_2$ Fragment Goat Anti-Human IgG, Fcγ fragment specific (Jackson ImmunoResearch, Cat#109-116-170)
-Goat anti-Human IgG Fc Highly Cross-Adsorbed Secondary Antibody, HRP (Invitrogen, Thermo Fisher, Cat#A18829)

Rabbit anti-6-His Tag Antibody HRP Conjugated (Bethyl Laboratories, Cat#A190-114P)
Rabbit anti-mouse IgG-HRP (Cedarlane, Cat#610-4302)

Polyclonal Rabbit SARS-CoV-2 Spike Antibody (Sino Biological, Cat#40589-T62)

Polyclonal Rabbit Anti-human CD3 (Dako, Cat#A0452)

Polyclonal Rabbit Anti-Iba-1 (Dako, Cat#019-19741)

Mouse SARS-CoV-2 Nucleocapsid MAb (R&D Systems, Cat#MAB10474, Clone 1035111)

Validation

Anti-EGFR NRCsdAb022 and anti-SARS-CoV-1 VHH-72 nanobodies were validated in this study. Anti-C. difficile toxin A A20.1 nanobody was validated in a previous study cited in the manuscript.

All commercial primary antibodies (described below) were validated by the manufacturers.

Polyclonal Rabbit SARS-CoV-2 Spike Antibody (Sino Biological, Cat#40589-T62). According to the site (https://www.sinobiological.com/antibodies/cov-spike-40589-t62): The antibody is produced in rabbits immunized with purified, recombinant SARS-CoV-2 spike protein (S1+S2 ECD) (Catalog#40589-V08B1; YP_009724390.1; Val16-Pro1213). The specific IgG is purified by protein A and SARS-CoV-2 spike affinity chromatography. It has specificity for SARS-CoV-2 coronavirus spike and cross-reactivity with SARS-CoV spike S1 (Cat# 40150-V08B1), SARS-CoV spike RBD (Cat# 40150-V08B2), SARS-CoV-2 spike S1 (Cat# 40591-V08H) and SARS-CoV-2 spike RBD (Cat# 40592-V05H). The antibody is validated for western blot and ELISA applications.

Polyclonal Rabbit Anti-human CD3 (Dako, Cat#A0452). According to the site (https://www.agilent.com/en/product/immunohistochemistry/antibodies-controls/primary-antibodies/cd3-(concentrate)-76133#specifications): Synthetic peptide from the intracellular part of the e-chain of human CD3 was coupled to bovine thyroglobulin and used for immunization to prepare rabbit anti-human CD3. The prep was affinity-isolated using immobilized CD3 peptide. The antibody is a pan-T cell marker for identification of T cells. It is well-suited for labeling reactive T cells in tissue with lymphoid infiltrates, and for classification of T-cell neoplasms. It has excellent specificity, high lot to lot consistency and been optimized for immunohistochemistry with validated protocols.

Polyclonal Rabbit Anti-Iba-1 (Dako, Cat#019-19741). According to the site (https://labchem-wako.fujifilm.com/us/product/detail/W01W0101-1974.html): Polyclonal rabbit anti-Iba-1 was raised against a synthetic peptide corresponding to the Iba-1 carboxy-terminal sequence, which is conserved among human, rat and mouse Iba-1 protein sequences. The polyclonal is purified by the antigen affinity chromatography from rabbit antisera. It has reactivity towards mouse, rat, human and other species Iba-1. Anti-Iba-1 is specifically reactive to microglia and macrophages and has been validated for Immunocytochemistry applications.

Mouse SARS-CoV-2 Nucleocapsid MAb (Cat#MAB10474, Clone 1035111). According to the site (https://www.rndsystems.com/products/sars-cov-2-nucleocapsid-mab-clone-1035111-1035111_mab10474): The source of the antibody is monoclonal mouse IgG2B clone #1035111, obtained by immunizing mice with Spodoptera frugiperda, Sf 21-derived SARS-CoV-2 nucleocapsid protein Met1-Ala419 (Accession # YP_009724397.2). The antibody is purified from hybridoma culture supernatant by protein A or G affinity chromatography. It detects SARS-CoV-2 nucleocapsid in direct ELISAs and Western blots. No cross-reactivity with MERS nucleocapsid

is observed in Western blots. The antibody is validated for detection of SARS-CoV-2 nucleocapsid protein in western blot and immunohistochemistry applications.

Eukaryotic cell lines

Policy information about cell lines and Sex and Gender in Research

Cell line source(s)	Commercial sources for the cell lines are: Vero E6 cells, ATCC Cat#CRL-1586; HEK293T, ATCC Cat#CRL-11268; HEK293T-hACE2, BEI Resources Cat#NR-52511.
Authentication	None of the cell lines used were authenticated.
Mycoplasma contamination	The cell lines were not tested for mycoplasma contamination.
Commonly misidentified lines (See ICLAC register)	No commonly misidentified cell lines were used in this study.

Animals and other research organisms

Policy information about studies involving animals; ARRIVE guidelines recommended for reporting animal research, and Sex and Gender in Research

Laboratory animals	For immunizations, four years old female llamas (Lama glama) were used.<br><br>For in vivo serum stability and efficacy studies, 6-7 weeks old, female LVG Golden Syrian hamsters (90-100 g) were used.
Wild animals	The study did not involve wild animals.
Reporting on sex	n/a
Field-collected samples	The study did not involve samples collected from the field.
Ethics oversight	Llama immunizations were conducted using protocols approved by the National Research Council Canada Animal Care Committee and in accordance with the guidelines set out in the OMAFRA Animals for Research Act, R.S.O. 1990, c. A.22.<br><br>For hamster studies, all animal procedures and animal husbandry were carried out in accordance to regulations and guidelines outlined under the Canadian Council on Animal Care and approved by the NRC Human Health Therapeutics Animal Care Committee.

Note that full information on the approval of the study protocol must also be provided in the manuscript.

Flow Cytometry

Plots

Confirm that:

- [ ] The axis labels state the marker and fluorochrome used (e.g. CD4-FITC).
- [ ] The axis scales are clearly visible. Include numbers along axes only for bottom left plot of group (a 'group' is an analysis of identical markers).
- [ ] All plots are contour plots with outliers or pseudocolor plots.
- [ ] A numerical value for number of cells or percentage (with statistics) is provided.

Methodology

Sample preparation	*Describe the sample preparation, detailing the biological source of the cells and any tissue processing steps used.*
Instrument	*Identify the instrument used for data collection, specifying make and model number.*
Software	*Describe the software used to collect and analyze the flow cytometry data. For custom code that has been deposited into a community repository, provide accession details.*
Cell population abundance	*Describe the abundance of the relevant cell populations within post-sort fractions, providing details on the purity of the samples and how it was determined.*
Gating strategy	*Describe the gating strategy used for all relevant experiments, specifying the preliminary FSC/SSC gates of the starting cell population, indicating where boundaries between "positive" and "negative" staining cell populations are defined.*

- [ ] Tick this box to confirm that a figure exemplifying the gating strategy is provided in the Supplementary Information.
